# PHF13 epigenetically activates TGFβ driven epithelial to mesenchymal transition

**DOI:** 10.1038/s41419-022-04940-4

**Published:** 2022-05-21

**Authors:** Yating Sun, Dan Li, Hongmei Liu, Yongye Huang, Fanyu Meng, Jiahao Tang, Zhanjun Li, Wanhua Xie

**Affiliations:** 1 The Precise Medicine Center, Department of Basic Medical College, Shenyang Medical College, Shenyang, 110034 China; 2 School of Pharmacy, Shenyang Medical College, Shenyang, 110034 China; 3 CAS Key Laboratory of Regenerative Biology, Guangzhou Institutes of Biomedicine and Health, Chinese Academy of Sciences, Guangzhou, 510530 China; 4 Research Unit of Generation of Large Animal Disease Models, Chinese Academy of Medical Sciences (2019RU015), Guangzhou, 510530 China; 5 College of Life and Health Sciences, Northeastern University, Shenyang, 110169 China; 6 Key Laboratory of Zoonosis Research, Ministry of Education, College of Animal Science, Jilin University, Changchun, 130062 China

**Keywords:** Transcriptional regulatory elements, Epithelial-mesenchymal transition

## Abstract

Epigenetic alteration is a pivotal factor in tumor metastasis. PHD finger protein 13 (PHF13) is a recently identified epigenetic reader of H3K4me2/3 that functions as a transcriptional co-regulator. In this study, we demonstrate that PHF13 is required for pancreatic-cancer-cell growth and metastasis. Integrative analysis of transcriptome and epigenetic profiles provide further mechanistic insights into the epigenetic regulation of genes associated with cell metastasis during the epithelial-to-mesenchymal transition (EMT) induced by transforming growth factor β (TGFβ). Our data suggest PHF13 depletion impairs activation of TGFβ stimulated genes and correlates with a loss of active epigenetic marks (H3K4me3 and H3K27ac) at these genomic regions. These observations argue for a dependency of TGFβ target activation on PHF13. Furthermore, PHF13-dependent chromatin regions are enriched in broad H3K4me3 domains and super-enhancers, which control genes critical to cancer-cell migration and invasion, such as SNAI1 and SOX9. Overall, our data indicate a functional and mechanistic correlation between PHF13 and EMT.

## Introduction

Epithelial-to-mesenchymal transition (EMT) is a cellular process that plays a central role in embryonic development, wound healing, and malignant tumor progression [1]. Abnormal activation of EMT confers multiple characteristics on carcinoma cells during cancer progression, involving stemness, invasiveness, and therapeutic resistance associated with high-grade malignancy [2–4].

The TGFβ pathway is one of the most well-characterized pathways that is known to induce EMT. In response, mesenchymal genes (N-cadherin, vimentin, fibronectin, etc.) and EMT-induced transcription factors (SNAI1, SNAI2, ZEB1, and TWIST) are activated. These EMT-TFs, in turn, repress epithelial genes and elevate the expression of mesenchymal genes. These widespread transcriptional changes are accompanied by complex epigenetic regulatory mechanisms, notably alterations in the post-transcriptional modification of histones. Interestingly, TGFβ treatment induces widespread alteration of histone modifications via increasing chromatin accessibility [5]. Furthermore, epigenetic-profiling studies have identified novel chromatin signatures, notably broad H3K4me3 domains and super-enhancers, that contribute to the initiation and development of cancers and provide potential targets for therapeutic intervention [6–8].

The broad H3K4me3 domains regulate embryonic development and human disease [8–12]. For example, the broad H3K4me3 domains are associated with the high transcriptional activity of tumor-suppressor genes in normal cells [6–8]. Interestingly, some studies have found newly formed broad H3K4me3 on oncogenes in cancer development [6, 13]. In addition, WDR5 and MLL4, which function as epigenetic writers to facilitate broad H3K4me3, are required to activate mesenchymal genes and EMT-TFs [8, 10, 14, 15]. However, the importance of the newly formed broad H3K4me3 in cancer progression (e.g., in the EMT process) remains unclear.

Enhancers, signed by active histone markers, including H3K27ac and H3K4me1, interact with promoters to form chromatin loops, resulting in transcriptional activation. The epigenetic landscape of transcription activators has identified a novel epigenetic signature termed “super-enhancers” (SEs), which strongly activate the expression of genes that control cell identity [16–18]. Oncogenic SEs, which are formed in cancers rather than normal tissues, play critical roles in various kinds of tumors [18]. Interestingly, oncogenic SEs are more sensitive to perturbation than typical enhancers and show potential therapeutic epigenetic targets for many malignant tumors [19–26]. In addition, widespread chromatin opening of enhancers has been documented in TGFβ-induced EMT [5, 27]. However, whether TGFβ-induced EMT coincides with changes in SE activity is still unknown.

PHF13 is a plant homeodomain (PHD) finger-containing protein that plays an essential role in modulating the cell cycle [28], DNA damage repair [29], and chromatin structure [28]. A recent study found that PHF13 specifically recognizes H3K4me2/3 and functions as a transcription coactivator to facilitate gene transcription [30–32]. Interestingly, PHF13 also interacts with EZH2, a core subunit of the polycomb repressive complex 2 (PRC2), on a particular chromatin region termed “bivalent,” which is co-occupied by the active histone marker H3K4me3 and repressive histone marker H3K27me3 [31]. Bivalent domains keep master regulators of cell fate transitions silent yet “poised” for rapid activation by extracellular signals, such as TGFβ [27, 33, 34]. However, the function of PHF13 on bivalent domains remains unclear. Highly expressed PHF13 has been observed in unresectable ovarian cancer and is associated with a lower survival rate [35]. However, its role and therapeutic targetability in human carcinomas remain unknown.

In this study, we demonstrate that PHF13-depleted pancreas ductal adenocarcinoma cells Panc-1 show a significantly decreased cell proliferation in vitro and in vivo. Moreover, cell culture-based assays reveal that PHF13 is required for TGFβ-induced EMT. Using the integrated transcriptomic and epigenetic analysis, we provide the first evidence that a subset of TGFβ-activated genes requires PHF13 to increase the activity of poised regions, broad H3K4me3 domains, and super-enhancers. Together, these findings suggest a novel epigenetic mechanism of PHF13 in promoting cancer-cell migration and identify PHF13 as a potential therapeutic target for human pancreatic cancer.

## Results

### High expression of PHF13 is associated with pancreatic adenocarcinoma

**Fig. 1:**
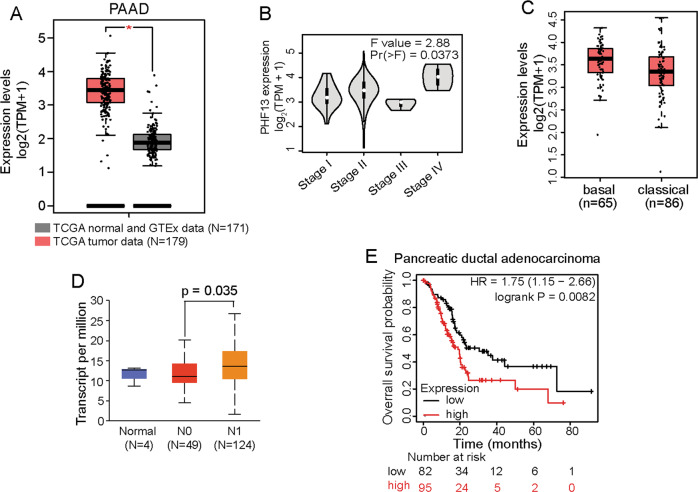
High expression of *PHF13* is associated with human cancer. **A** Boxplot showing TPM-normalized expression of *PHF13* in normal tissues and pancreatic adenocarcinoma (PAAD). The results were generated by the online tool: GEPIA [73]. Given that there are only 4 normal pancreatic samples in TCGA data, we combined them with the pancreatic samples in GTEx data. **B** Violin plot showing the expression of PHF13 at different clinical stages in patients with PAAD. The data was generated using the GEPIA tool [73]. **C** Boxplot comparing the TPM-normalized expression of PHF13 between basal-like and classical subtypes. The data was generated using the GEPIA2 tool [73]. **D** Boxplot showing normalized expression of PHF13 in normal pancreatic tissues (Normal), pancreatic tumors with no regional lymph node metastasis (N0), and pancreatic tumors with metastases in 1 to 3 axillary lymph nodes (N1) in the TCGA cohort. N number of patients. The result was generated from the online tool UALCAN [74]. **E** Kaplan–Meier plot showing the overall survival of patients based on *PHF13* expression. Data is generated from the webserver (http://kmplot.com/analysis/) [75]. HR hazard ratio, CI confidence interval.

To understand the role of PHF13 in pancreatic cancer, we investigated its expression pattern in human tumors, compared with normal tissues, and found an elevated expression of *PHF13* in pancreatic adenocarcinoma (Fig. 1A). Moreover, highly expressed *PHF13* was significantly correlated with high-grade pancreatic cancer (Fig. 1B). In addition, *PHF13* showed much higher expression in the basal-like subtype of pancreatic cancer that is characterized by aggressive activity, undifferentiated histopathology, and worse prognosis, compared to the classical subtype (Fig. 1C). The expression of *PHF13* was also significantly higher in pancreatic cancer with metastases in axillary lymph nodes (Fig. 1D). Importantly, Kaplan–Meier analysis demonstrated that high *PHF13* expression significantly worsened survival rates in patients with pancreatic ductal adenocarcinoma (Fig. 1E). Together, these results demonstrate the correlation between PHF13 expression and patient outcomes, indicating a potential oncogenic role for PHF13 in pancreatic cancer.

### Perturbation of PHF13 suppresses pancreatic cancer cell proliferation in vitro and in vivo

**Fig. 2:**
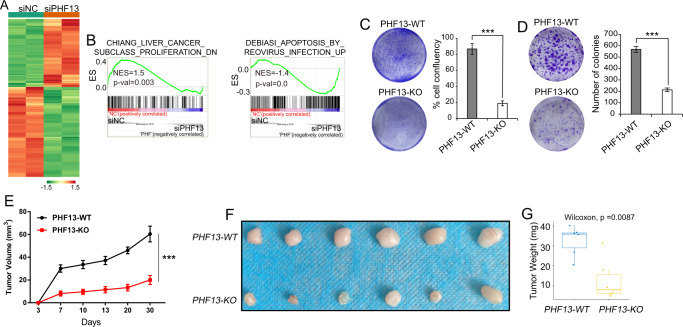
Perturbation of *PHF13* suppresses pancreatic cancer cell proliferation in vitro and in vivo. **A** Heatmap showing the differential expressed genes following PHF13 depletion in pancreatic adenocarcinoma cells Panc-1. The color key was shown at the bottom of the graph. The significantly regulated genes were selected based on**:** the absolute value of log2-fold change > 0.58, the p-adj < 0.05 **B** GSEA showing the significant enrichment of gene sets associated with cell proliferation (left panel), and apoptosis (right panel). NES, normalized enrichment score. **C** Cell proliferation assay for PHF13-WT and PHF13-KO Panc1. Image of crystal violet staining (left panel). The confluency of stained cells in each well was calculated by ImageJ (right panel). The data are shown as the mean ± SD. ***p < 0.001, calculated by two-side *t* test. all experiments were performed in triplicate. **D** Colony-forming assay for PHF13-WT and PHF13-KO Panc1 cells. The left panel indicates the image of crystal violet staining for the colonies. ImageJ was utilized to calculate the numbers of stained colonies in each well (right panel). Biological triplicate experiments were performed. The data are represented as the mean ± SD. ***p < 0.001, calculated by two-side *t* test. **E** Representative graphs showing the size of tumors formed by transplanting *PHF13*-WT and *PHF13*-KO Panc1 cells into the right groin of the mouse to establish an orthotopic model. Error bars represent the standard error of the mean between the six biological replicates. ***p < 0.001, calculated by two-side *t* test. **F** The pictures showed the tumors formed by transplanting PHF13-WT and PHF13-KO Panc-1 cells. The xenografts were harvested at 30 days. **G** boxplot showing the weight of tumors implanting PHF13-WT and PHF13-KO Panc1 cells for 30 days. *P* value was calculated by the unpaired Wilcoxon–Mann–Whitney Test.

To determine the role of PHF13 in human pancreatic cancers, we performed mRNA-seq upon siRNA-mediated depletion of PHF13 in Panc-1 (Supplementary Fig. 1A). In support of the role of PHF13 in regulating mitosis [28], GSEA analysis of the differential expressed genes upon PHF13 depletion (Fig. 2A, Supplementary Table 3) revealed a significantly negative enrichment of the cell proliferation-related pathway (Fig. 2B, left panel) and overrepresentation of apoptosis-associated gene set in PHF13-depleted cells (Fig. 2B, right panel). The in vitro proliferation assay further verified the negative effect of PHF13 depletion on Panc-1 cell growth (Supplementary Fig. 1B).

We next sought to substantiate these findings in an in vivo system. For this purpose, we utilized the CRISPR/Cas9 gene-editing tool to induce PHF13 mutation in Panc-1 cells. Two guide RNAs were designed to specifically target exon 3 of the PHF13 gene (Supplementary Fig. 1C). Allele mutation of PHF13 in single-cell clone was screened by genotyping PCR and Sanger sequencing (Supplementary Fig. 1D, E). PHF13 knockout consistently and significantly suppressed the proliferation and colony formation of Panc-1 cells (Fig. 2C, D and Supplementary Fig. 1F). Notably, PHF13 deletion significantly reduced the size and weight of tumors formed by Panc-1 cells in vivo (Fig. 2E–G and Supplementary Table 4). Thus, in vitro and in vivo data provide further support for the role of PHF13 in controlling pancreatic-cancer-cell proliferation.

### Depletion of PHF13 impairs TGFβ-induced EMT

**Fig. 3:**
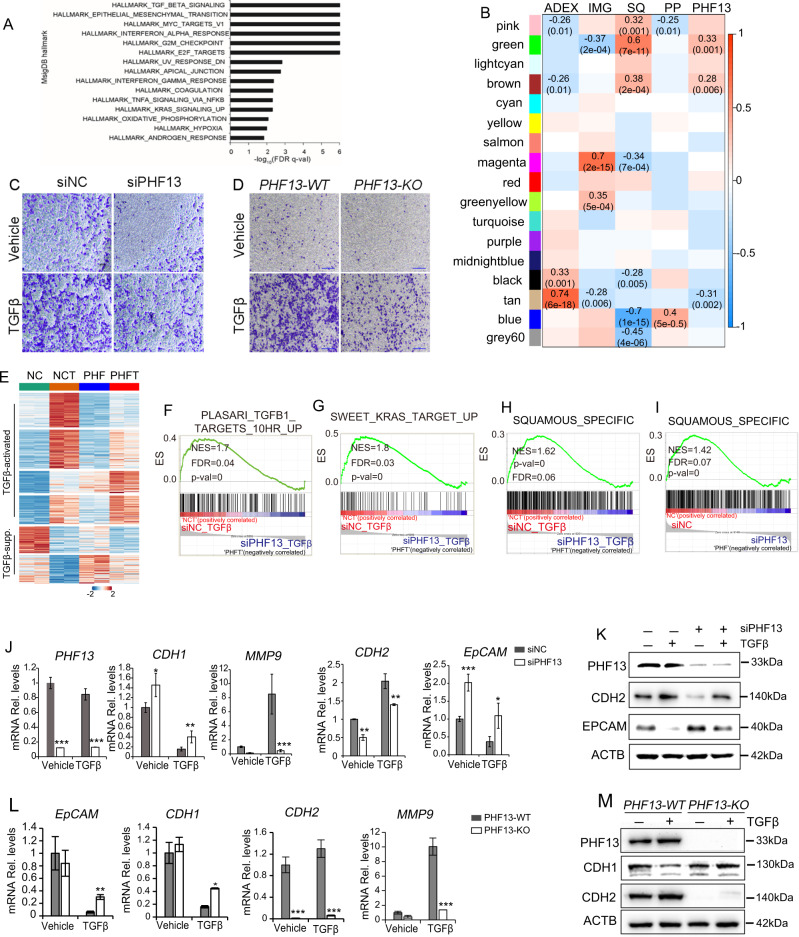
PHF13 is required for TGFβ-induced EMT. **A** The top 15 significantly enriched MSigDB Hallmark gene sets via GSEA for the transcriptomic profiles of PHF13-depleted and control cells. FDR false discovery rate. **B** Heatmap showing the statistical significance of correlations between each subtype of pancreatic cancers, PHF13 expression levels, and gene modules identified by WGCNA. Bailey et al. determined the four molecular subtypes of pancreatic cancers: pancreatic progenitor (PP), immunogenic (IMG), aberrantly differentiated endocrine exocrine (ADEX), and squamous (SQ) [39]. The significantly correlated cells contain Student’s asymptotic *P* values and Pearson correlations. Transwell migration assay showing a significant decrease in migration potential of TGFβ-treated cells in response to PHF13 depletion (**C**) or CRISPR/Cas9-mediated knockout (**D**). The migrated cells were stained with 0.1% (w/v) crystal violet (scale bar 100 μm). The experiment was performed in duplicate. **E** Heatmaps showing the significantly regulated genes upon TGFβ treatment in Panc-1 cells. K-means clustering (*K* = 6) was performed based on the normalized expression level using the option scale = “row” during the following conditions: Control (NC), Control with TGFβ treatment (NCT), PHF13 knockdown (PHF), and TGFβ-treated cells depleted for PHF13 (PHFT). The color scale bar shows the row-wise normalized value. GSEA showing a significant decrease in the enrichment of TGFβ targets (**F**), KRAS targets (**G**), and squamous-specific genes (**H**, **I**) in PHF13-depleted Panc-1 cells. Squamous-specific genes were generated from the published data [39]. **J** RT-qPCR analysis of *PHF13* knockdown efficiency, epithelial (*CDH1* and *EpCAM*), and mesenchymal (*CDH2* and *MMP9*) markers in Panc-1 cells in response to TGFβ treatment and PHF13 depletion. The data represent the mean ± SD. *p < 0.05; **p < 0.01; ***p < 0.001, and calculated by two-side *t* test. **K** western blots showing the protein level of PHF13, CDH2, and EpCAM in Panc-1 following TGFβ induction and PHF13 knockdown. The level of ACTB was set as a loading control. **L** RT-qPCR analysis of epithelial (*CDH1* and *EpCAM*) and mesenchymal (*CDH2* and *MMP9*) markers in *PHF13*-WT and *PHF13*-KO Panc1 following TGFβ treatment. **M** Western blot analysis of the protein level of PHF13, CDH1, and CDH2 in the given samples. ACTB was set as a loading control.

Interestingly, GSEA further identified significant enrichment of cell migration-associated pathways involved in TGFβ signaling and EMT via comparing the transcriptomic profiles of PHF13-depleted and control Panc-1 (Fig. 3A). To confirm the findings in vitro, we analyzed the public available RNA-seq data of human pancreatic cancers to identify the differential expressed genes between PHF13 highly and lowly expressed pancreatic cancers (Supplementary Fig. 2A–C and Supplementary Table 5). Significantly, GO enrichment analysis of the differentially expressed genes identified the extracellular matrix organization pathway, associated with cell migration (Supplementary Fig. 2D–F). Moreover, weighted gene co-expression network analysis (WGCNA) was performed to determine the correlations between the expression of PHF13 and the subtypes of pancreatic cancers. We demonstrated that the SQ trait-associated “green” and “brown” gene modules, which displayed significant enrichment of pathways involved in the extracellular matrix, tissue development, and cell junctions (Supplementary Fig. 2H, I), were significantly related to the high expression level of PHF13 (Fig. 3B). Thus, those findings uncovered PHF13 as a potential regulator of cell migration-related gene programs in human pancreatic cancer.

To confirm the potential role of PHF13 in controlling tumor metastasis, we investigated the influence of PHF13 depletion on TGFβ-induced EMT. Upon TGFβ stimulation, Panc-1 cells acquired a more pronounced mesenchymal phenotype than the PHF13-deleted cells (Supplementary Fig. 2J). In addition, the transwell migration assay revealed a significantly decreased migration potential in PHF13-depleted and -knockout Panc-1 (Fig. 3C, D). Furthermore, we performed a transcriptome-wide analysis of the effects of PHF13 depletion on TGFβ-regulated genes. siRNA-mediated depletion of PHF13 was confirmed by RT-qPCR and western blot analysis (Fig. 3J, K). K-means clustering coupled with a genome-wide pairwise comparison of heatmaps further revealed that the depletion of PHF13 negatively or positively regulated a large group of TGFβ-controlled genes (Fig. 3E and Supplementary Table 3). GSEA confirmed significantly opposite effects of PHF13 depletion on TGFβ-regulated genes (Fig. 3F; Supplementary Fig. 2K, L). Interestingly, PHF13 depletion significantly downregulated the targets of KRAS (3G), which is frequently activated in most pancreatic adenocarcinomas and is known to be an essential driver of human cancer [36–38]. Moreover, the preferentially expressed genes in the most aggressive SQ subtype of pancreatic cancers [39] were negatively enriched in PHF13-depleted cells (Fig. 3H, I). Furthermore, using RT-qPCR and western blot assay, we confirmed that PHF13 perturbation significantly upregulated epithelial markers (*CDH1* and *EpCAM*) and downregulated mesenchymal markers (*CDH2* and *MMP9*) (Fig. 3J–M). In contrast, re-expressing PHF13 in PHF13-KO Panc-1 resulted in a significant reduction of CDH1 and gain of CDH2 (Supplementary Fig. 2M). These results suggest that PHF13 plays an essential role in controlling pancreatic-cancer-cell metastasis through maintaining and directing EMT-associated gene expression.

### PHF13 is required for TGFβ-mediated activation of enhancers and promoters

**Fig. 4:**
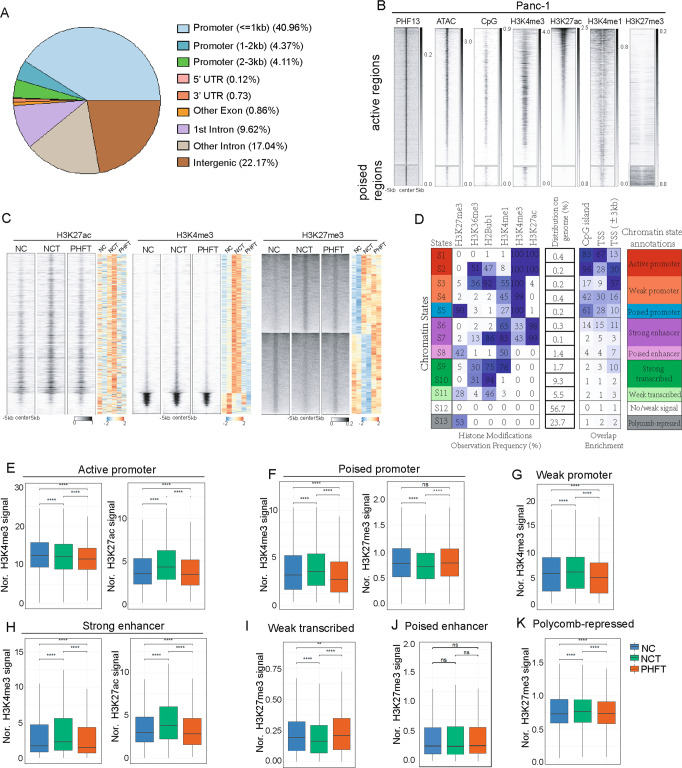
PHF13 is required for the TGFβ-mediated activation of enhancers and promoters. **A** Distribution of PHF13 occupancy on distinct chromatin elements. **B** Heatmaps show the occupancy of chromatin assembly (ATAC-seq), CpG island, H3K4me3, H3K27ac, H3K4me1, and H3K27me3 surrounding the center of PHF13 peaks (±5 kb). The color key of each heatmap is shown on the right side. **C** Pairwise heatmaps show the normalized density and distribution of the differentially changed H3K27ac, H3K4me3, and H3K27me3 in siNC (NC), siNC + TGFβ (NCT), and siPHF13 + TGFβ(PHFT) Panc1 cells. The differentially changed chromatin sites upon TGFβ treatment were identified by DiffBind (Supplementary Fig. 3B). The region flanking ±5 kb of each peak center is shown. The right color heatmap shows the row-wise normalized signal of H3K27ac, H3K4me3, and H3K27me3. **D** ChromHMM was used to learn and characterize chromatin states in Panc1 cells. The first table from left to right shows the emission parameters determined de novo with ChromHMM from the given chromatin markers over the entire genome. The second table indicates the distribution of each characterized chromatin state on the genome. The third table displays the relative fold enrichment of each chromatin state on several chromatin elements, including CpG islands, TSS, and promoters (surrounding TSS ± 3 kb). Chromatin annotations for each state are shown on the rightest table. Boxplots compare the occupancy of H3K4me3, H3K27ac, and H3K27me3 at the genomic regions corresponding to the identified states, including active promoter (**E**), poised promoter (**F**), weak promoter (**G**), strong enhancer (**H**), weak transcribed (**I**), poised enhancer (**J**), and polycomb-repressed (**K**) chromatin regions, in siNC (NC), siNC + TGFβ (NCT), and siPHF13 + TGFβ(PHFT) Panc1 cells. *p* value was calculated using the unpaired Wilcoxon–Mann–Whitney Test. ns nonsignificant difference; **p value greater than 0.001 but less than 0.01; ****p value less than 0.0001.

To determine the epigenetic function of PHF13 in gene expression, we performed ChIP-seq analysis of PHF13. We further overlapped the accessible sites (ATAC-seq) with the PHF13-bound sites to preferentially select the PHF13-occupied regions (Supplementary Fig. 3A). Interestingly, the PHF13-occupied regions were mainly distributed on TSS-proximal promoters and distal enhancers (Fig. 4A). in support of previous findings [31], heatmap analysis of the occupancy of H3K4me3, H3K27me3, and H3K27ac surrounding PHF13-bound regions revealed that PHF13 was enriched in the active and poised chromatin regions (Fig. 4B). Next, we analyzed ChIP-seq profiles of H3K4me3, H3K27me3, and H3K27ac to uncover the role of PHF13 in the TGFβ-triggered alterations in chromatin activity. Upon stimulation of TGFβ, the regions displaying differential occupancy of H3K4me3, H3K27me3, and H3K27ac in Panc-1 were identified via DiffBind analysis (Supplementary Fig. 3B). The pairwise comparison analysis of heatmaps further revealed that the occupancy of H3K4me3 and H3K27ac were decreased, whereas the occupancy of H3K27me3 was increased in a subset of TGFβ-activated regions in response to PHF13 depletion in TGFβ-treated cells compared to cells with TGFβ treatment alone (Fig. 4C).

To determine which chromatin states are highly dependent on PHF13, chromatin segmentation was performed, and 13 specific chromatin states were identified via ChromHMM [40] analysis of the H3K27me3, H3K36me3 [41], H2Bub1, H3K4me1 [42], H3K4me3, and H3K27ac occupancy profiles in Panc-1 cells. Based on the enrichment of each chromatin state on the CpG island, TSS, and TSS-proximal promoter (TSS ± 3 kb), the 13 chromatin states fit into nine functional classes: active promoter, weak promoter, poised promoter, strong enhancer, poised enhancer, strong transcribed, no/weak transcribed, no/weak signal, and polycomb-repressed regions (Fig. 4D). Boxplot further compared the occupancy of H3K4me3, H3K27ac, and H3K27me3 at each chromatin state. In general, TGFβ treatment in Panc-1 resulted in increased activity of each chromatin state except for poised enhancers and polycomb-repressed regions, showing significantly increased occupancy of H3K4me3 and H3K27ac and decreased occupancy of H3K27me3 compared to the control cells (Fig. 4E–I). In comparison to TGFβ treatment alone, PHF13 depletion in TGFβ-treated Panc-1 caused opposite effects (Fig. 4E–I). However, there were no visible changes in poised enhancers (Fig. 4J). In the polycomb-repressed regions, the occupancy of H3K27me3 was significantly increased in TGFβ-induced cells compared to the control cells, while slightly decreased in PHF13-depleted TGFβ-treated cells compared to cells with TGFβ treatment alone (Fig. 4K). Thus, we propose that depletion of PHF13 impairs TGFβ-triggered epigenetic activation.

### The expression of TGFβ-stimulated poised genes is highly dependent on PHF13

**Fig. 5:**
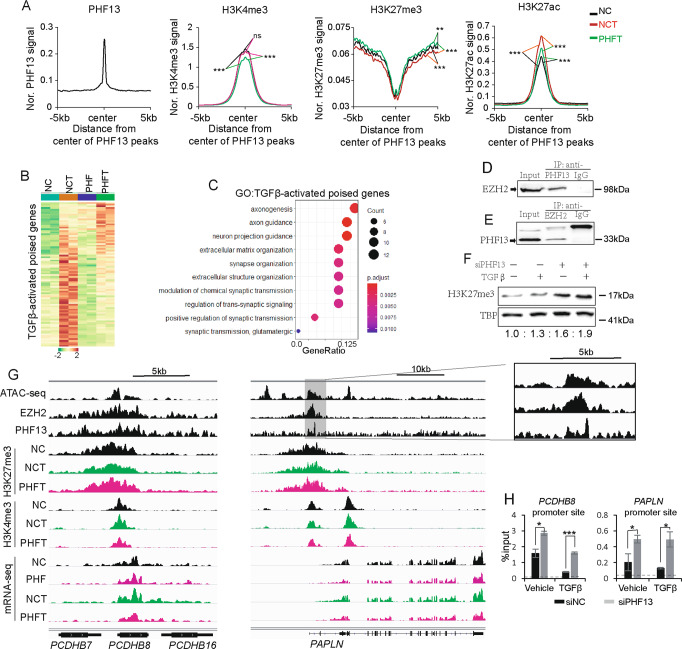
The expression of TGFβ-stimulated poised genes is highly dependent on PHF13. **A** Aggregate profiles show the occupancy of H3K4me3, H3K27me3, and H3K27ac surrounding the center of PHF13-occupied poised chromatin regions (±5 kb) in siNC, siNC + TGFβ (NCT), and siPHF13 + TGFβ (PHFT) Panc-1 cells. *P* values were calculated by Wilcoxon–Mann–Whitney Test. ns nonsignificant difference; **0.01 < *p* < 0.001; ***p < 0.001. **B** Heatmaps show gene expression of TGFβ-activated poised genes in siNC (NC), siPHF13 (PHF), siNC + TGFβ (NCT), and siPHF13 + TGFβ (PHFT) Panc1 cells. The color scale bar shows the row-wise normalized expression signal. **C** GO analysis of the TGFβ-activated poised genes. The top 10 significantly enriched gene terms were shown. Western blot analysis of nuclear extract inputs, anti-PHF13 IP (**D**), anti-EZH2 IP (**E**), and anti-IgG IP from Panc1 nuclear extract. **F** Western blot showing the level of H3K27me3 in the given samples. The protein TBP was set as a loading control. The down numbers indicated the normalized signal of H3K27me3. The signal of each band was measured using ImageJ. The signal of H3K27me3 was normalized to TBP and represented as fold change relative to the control cells. **G** Genome browser tracks the normalized signal of chromatin assembly (ATAC-seq), EZH2, PHF13, H3K27me3, H3K4me3 occupancy, and mRNA-seq data at *PCDHB8* an*d PAPLN lo*cus from siNC (NC), siPHF13 (PHF), siNC + TGFβ (NCT), siPHF13 + TGFβ (PHFT) Panc1 cells. **H** ChIP-qPCR analysis of H3K27me3 occupancy near the TSS of the *PCDHB8* and *PAPLN* genes in siControl treated, PHF13-depleted, TGFβ-treated, and PHF13-depleted TGFβ-treated Panc-1 cells. *p* value was calculated using a two-side *t* test. *p < 0.05; ***p < 0.001. ChIP-qPCR for IgG was set as a negative control. The dotted line indicated the average signal of IgG.

Given the essential role of the poised-chromatin state in cell fate determination [43], we further investigated the function of PHF13 on poised-chromatin regions in TGFβ-induced EMT. Aggregate profile analysis of H3K4me3, H3K27me3, and H3K27ac surrounding PHF13-occupied poised regions suggested a significantly increased occupancy of H3K27ac and decreased occupancy of H3K27me3 in TGFβ-treated cells relative to the control cells, whereas PHF13 depletion in TGFβ-treated cells significantly decreased H3K4me3 and H3K27ac occupancy and increased H3K27me3 occupancy compared to cells with TGFβ treatment alone (Fig. 5A). Pairwise comparison of heatmaps showed that a large fragment of TGFβ-stimulated poised genes was downregulated upon PHF13 depletion (Fig. 5B, Supplementary Table 3), supporting the alterations in the occupancy of histone modifications (Supplemental Fig. 4A). GO analysis of those genes revealed significant enrichment of pathways controlling the extracellular matrix and axonogenesis (Fig. 5C). Consistent with previous findings [31], endogenous co-immunoprecipitation (IP) assays showed that immunoprecipitation of PHF13 caused a co-IP of EZH2 (Fig. 5D) and vice versa (Fig. 5E). Interestingly, western blot analysis in Panc-1 showed a visibly increased level of H3K27me3 upon PHF13 depletion (Fig. 5F). This observation was mirrored by depleting PHF13 in HCT116 and Bel-7404 cells (Supplementary Fig. 4B). Consistently, boxplot analysis confirmed the significantly increased occupancy of H3K27me3 in PHF13-depleted and TGFβ-treated cells (Supplementary Fig. 4C). The effect of PHF13 depletion on H3K27me3 occupancy was confirmed at individual PHF13-dependent and TGFβ-stimulated poised genes (*PCDHB8* and *PAPLN*) via the genomic browser and ChIP-qPCR analysis (Fig. 5G, H). Thus, the data suggest that PHF13 is required for TGFβ-induced resolution of the poised states to active states.

### TGFβ-activated broad H3K4me3 domains are sensitive to PHF13 depletion

**Fig. 6:**
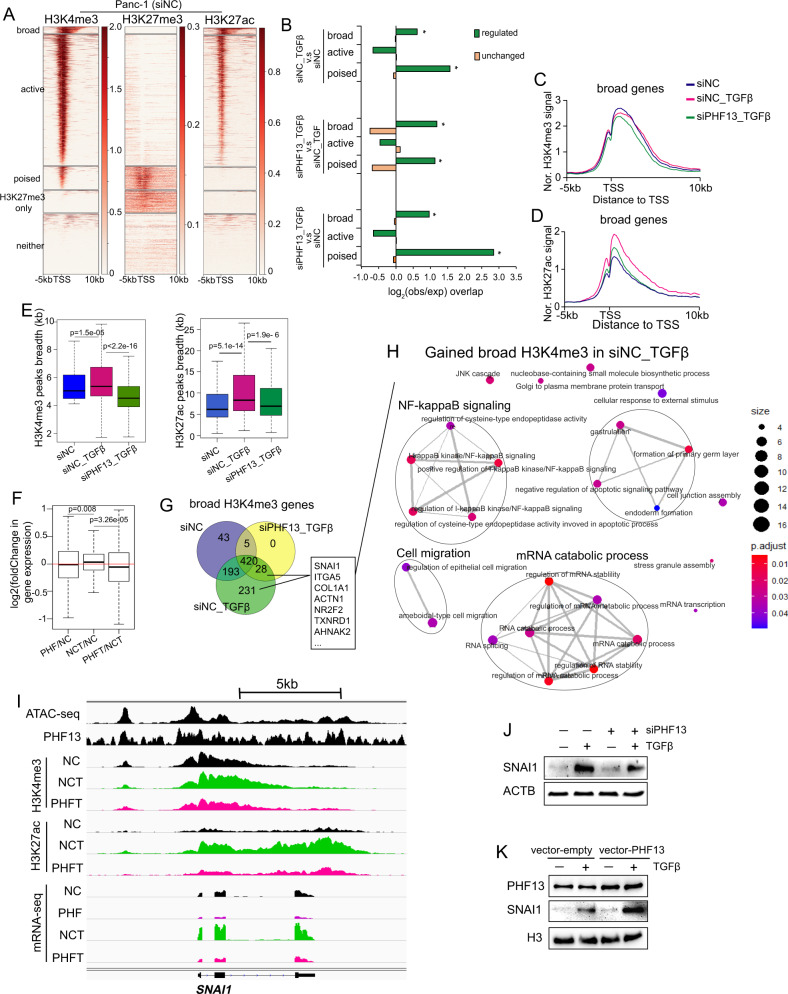
The broad H3K4me3 domain in TGFβ-induced EMT is highly dependent on PHF13. **A** The heatmaps show H3K4me3, H3K27me3, and H3K27ac on the regions flanking −5 kb to 10 kb around the TSS of all genes in Panc1 cells. All TSS were clustered based on the occupancy of the given histone modifications: the broad H3K4me3 occupied TSS (broad, defined based on Supplementary Fig. 5A), the typical H3K4me3 occupied TSS (active), H3K4me3 and H3K27me3 co-occupied TSS (poised), only H3K27me3-occupied TSS (H3K27me3 only), and non-histone modifications marked TSS (neither). The color key at the right side of each heatmap indicates the occupied density. **B** Observed/Expected enrichment of regulated and unchanged H3K4me3 occupied sites calculated from siNC_TGFβ versus siNC (up panel), siPHF13_TGFβ versus siNC_TGFβ (middle panel), and siPHF13_TGFβ versus siNC (down panel) at “broad”, “active”, and “poised” TSS regions. Aggregate profiles compare H3K4me3 (**C**) and H3K27ac (**D**) occupancy in siNC, siNC_TGFβ, and siPHF13_TGFβ Panc-1 cells at the regions from upstream 5 kb to downstream 10 kb of the broad H3K4me3-occupied TSS. **E** Boxplot compares the length of the broad H3K4me3 and H3K27ac peaks in siNC, siNC_TGFβ, and siPHF13_TGFβ Panc-1 cells. The unpaired Wilcoxon–Mann–Whitney Test was used to calculate *p* values. **F** Boxplot compares the log_2_-fold changes in the expression of broad H3K4me3-occupied genes in the following conditions: siPHF13 versus siNC (PHF/NC), siNC + TGFβ versus siNC (NCT/NC), and siPHF13 + TGFβ versus siNC + TGFβ (PHFT/NCT). **G** Venn diagram displaying the numbers of overlap and unique broad H3K4me3-occupied genes between siNC, siNC_TGFβ, and siPHF13_TGFβ Panc-1 cells. The broad H3K4me3-occupied genes in various conditions were obtained according to Supplementary Fig. 5A. **H** GO analysis of the TGFβ-gained broad H3K4me3-linked genes (correlated to Fig. 6G, the “231” and “28” genes). The enrichment map displays the connection of the top 25 significantly enriched gene sets. Four functional biological modules have been identified, including NF-kappaB signaling, cell migration, mRNA process, and endoderm formation. **I** Genome browser tracks the normalized signal of chromatin assembly (ATAC-seq), PHF13, H3K27ac, H3K4me3 occupancy, and mRNA-seq data at *SNAI1* locus from siNC (NC), siPHF13 (PHF), siNC + TGFβ (NCT), siPHF13 + TGFβ (PHFT) Panc-1 cells. **J** Western blot analysis shows the expression of SNAI1 in Panc-1 cells following TGFβ treatment and PHF13 depletion. **K** Western blots analysis shows the expression of SNAI1 in PHF13-overexpressed Panc-1 cells.

To investigate the epigenetic function of PHF13 on TSS-proximal regions, we divided TSS-proximal promoters into five clusters based on histone modifications as previously reported [44]: broad H3K4me3 occupied (broad), typical H3K4me3 occupied (active), H3K4me3/H3K27me3 co-occupied (poised), only H3K27me3 occupied (H3K27me3 only), and no histone markers (neither) (Fig. 6A). Interestingly, the chromatin sites, which displayed differential occupancy of H3K4me3 in each condition, were significantly enriched in the broad H3K4me3 and poised chromatin regions (Fig. 6B). In support of this observation, the upregulated genes upon TGFβ stimulation were enriched in “poised” and “broad” clusters (Supplementary Fig. 5A), whereas most of the two cluster genes were downregulated upon PHF13 depletion (Supplementary Fig. 5B, C).

Given the importance of broad H3K4me3 domains in cell fate determination and human disease [8, 10, 11, 44], we further examined the role of PHF13 in controlling the broad H3K4me3 domain in TGFβ-induced EMT. Therefore, we selected the broad-H3K4me3-occupied genes in untreated, TGFβ-induced, as well as PHF13-depleted TGFβ-treated Panc-1 cells, as in the previous report [44] (Supplementary Fig. 5D, Supplementary Table 3). Interestingly, aggregate profile and boxplot analysis revealed that the H3K4me3 and H3K27ac peaks surrounding TSS of broad H3K4me3-linked genes were broadened in TGFβ-treated cells compared to the control cells, whereas narrowed in PHF13-depleted TGFβ-stimulated cells relative to cells treated with TGFβ alone (Fig. 6C–E), which corresponded to the downregulation of those genes following PHF13 depletion (Fig. 6F). A similar effect was observed in the typical active regions, but not stronger than that in the broad H3K4me3 regions (Supplementary Fig. 5E). Furthermore, Venn diagram was performed to select the TGFβ-activated and broad H3K4me3-linked genes (231 + 28 genes showing broader H3K4me3 peaks in TGFβ-treated cells compared to the control) in Panc-1 (Fig. 6G). GO analysis further revealed that those selected genes were significantly enriched in pathways involved in NF-kappaB signaling, cell migration, and the mRNA catabolic process (Fig. 6H). Notably, we confirmed this finding in the individual example of PHF13-bound and TGFβ-activated gene *SNAI1*, whose expression was dependent on PHF13 and highly associated with cancer metastasis (Fig. 6I–K). Furthermore, we compared the transcriptomic profiles of PHF13-depleted and control Panc-1 using the SNAI1-regulated gene set collection in LNCaP [45] and LS174T [46] cells. GSEA revealed a significant overrepresentation of the SNAI1-suppressed gene set and negative enrichment of the SNAI1-dependent gene set in PHF13-depleted cells (Supplementary Fig. 5F). ChIP-seq profiles showed a visibly decreased occupancy of H3K4me3 and H3K27ac at the promoter and enhancer of the SNAI1 target *MMP2*, which corresponded to the downregulation of *MMP2* in PHF13-depleted TGFβ-treated cells compared to cells treated with TGFβ alone (Supplementary Fig. 5G). Thus, the data suggest that PHF13 is essential in facilitating the transcription of TGFβ-activated oncogenes because it maintains the establishment of broad H3K4me3 domains.

Given that our data provide the first evidence that TGFβ selectively elevates the expression of oncogenes by controlling the broad H3K4me3 domains, we re-analyzed the public data in normal mouse mammary gland NMuMG cells to avoid any discrepancies [5]. Similarly, the TGFβ-induced and broad H3K4me3-linked genes in NMuMG cells were selected (Supplementary Fig. 5H, I), which were significantly enriched in pathways involved in cell adhesion, differentiation, and the TGFβ signaling (Supplementary Fig. 5J). Consistently, aggregate profiling analysis confirmed that TGFβ treatment resulted in a widespread increase of H3K4me3, H3K27ac, and H3K4me1 occupancy surrounding TSS of the TGFβ-triggered broad H3K4me3-linked genes (Supplementary Fig. 5K) but not on the random controls (Supplementary Fig. 5L). Thus, the role of TGFβ in controlling broad H3K4me3 domains is highly conserved across species.

### PHF13 is required for the increased activity of a subset of super-enhancers in TGFβ-induced EMT

**Fig. 7:**
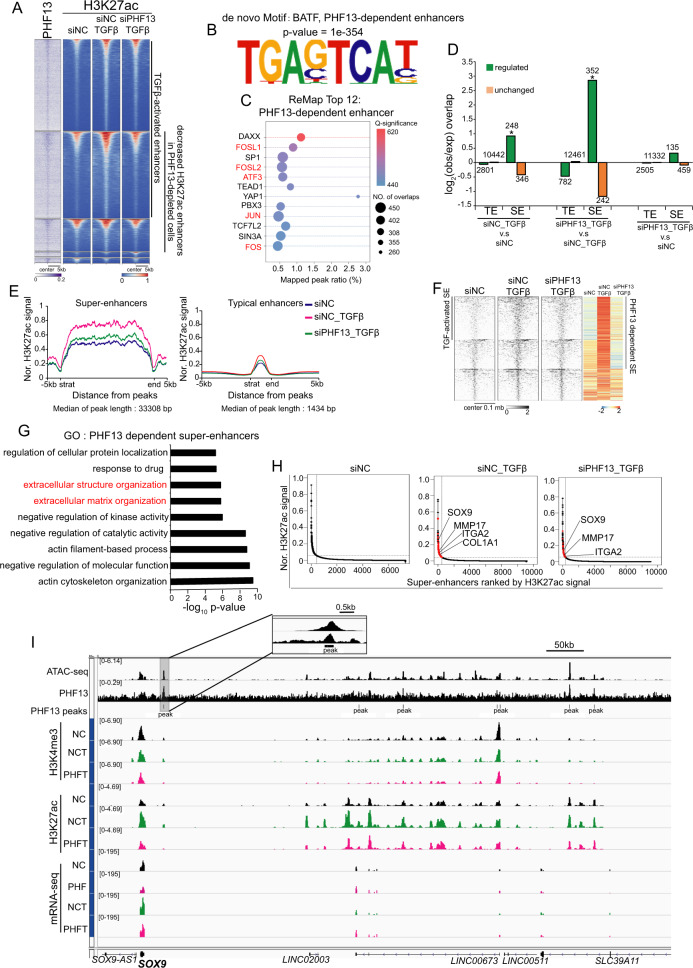
PHF13 is required for the increased activity of a subset of super-enhancers in TGFβ-induced EMT. **A** Pairwise heatmaps show the occupancy and distribution of PHF13 and H3K27ac over differential changed enhancers in siNC, siNC + TGFβ, and siPHF13 + TGFβ Panc-1 cells. Enhancers clustered according to the DiffBind analysis results (Supplementary Fig. 6A). TGFβ-activated enhancers, increased in siNC + TGFβ compared to siNC; PHF13-dependent enhancers, decreased in siPHF13 + TGFβ compared to siNC + TGFβ. The region flanking ±5 kb of each enhancer center is shown. The color scale bar shows the row-wise normalized signal of H3K27ac. **B** The de novo motif sequence of PHF13-dependent enhancers. DNA sequence-based motif analysis was performed on the PHF13-dependent enhancers (the 1201 PHF13 peaks identified in Supplementary Fig. 6B). The most significantly enriched motif is shown here. **C** ReMap analysis shows the top 12 transcription factors that hit PHF13-dependent enhancers. **D** Observed/Expected enrichment of regulated and unchanged H3K27ac occupied sites calculated from siNC_TGFβ versus siNC (left panel), siPHF13_TGFβ versus siNC_TGFβ (middle panel), and siPHF13_TGFβ versus siNC (right panel) at TEs and SEs. **E** Metagene profiles show the normalized signal of H3K27ac at typical enhancers (TE) and super-enhancers (SE) in siNC, siNC + TGFβ, and siPHF13 + TGFβ Panc-1 cells. The length of enhancers (from “start” to “end”) was calculated relative to the median length (TE, 1.4 kb; SE, 33 kb). **F** Pairwise heatmaps show the normalized density and distribution of H3K27ac over SEs in siNC, siNC + TGFβ, and siPHF13 + TGFβ Panc-1 cells. SEs were clustered using *K*-means (*K* = 3) based on the normalized occupancy of H3K27ac. The regions flanking ±100 kb of each SEs center are shown. The right color heatmap shows the row-wise normalized signal of H3K27ac and highlights the PHF13-dependent and TGFβ-activated SEs identified by *K*-means clustering. **G** GO analysis of PHF13-dependent SEs associated genes. The GREAT tool was used to predict SE annotated genes. The top 10 significantly enriched gene terms are shown. **H** Ranking of enhancers based on the normalized signal of H3K27ac in siNC, siNC + TGFβ, and siPHF13 + TGFβ Panc-1 cells. SEs are annotated by a GREAT online tool. The positions of SEs associated with *SOX9*, *MMP17*, *ITGA2*, and *COL1A1*, as well as the unique SEs in siNC + TGFβ or siPHF13 + TGFβ Panc-1 cells, are highlighted (red points). **I** Genome browser tracks the normalized signal of chromatin assembly (ATAC-seq), PHF13, H3K27ac, H3K4me3 occupancy, and mRNA-seq data at *SOX9* locus from siNC (NC), siPHF13 (PHF), siNC + TGFβ (NCT), siPHF13 + TGFβ (PHFT) Panc1 cells. One of the enhancers has been zoon out.

To further explain the changes in gene expression, we investigated alterations in enhancer activity through DiffBind analysis of H3K27ac occupancy (Supplementary Fig. 6A). The genome-wide pairwise comparison further revealed a decreased occupancy of H3K27ac at a large fragment of TGFβ-activated enhancers in PHF13-depleted TGFβ-treated cells compared to cells treated with TGFβ alone (Fig. 7A). Furthermore, Venn diagram analysis was utilized to select the PHF13-dependent enhancers (Supplementary Fig. 6A, B). The Genomic Regions Enrichment of Annotations Tool (GREAT) analysis revealed that the PHF13-dependent enhancers were significantly enriched in pathways controlling the extracellular matrix, cell migration, and EMT (Supplementary Fig. 6C). In addition, a de novo motif analysis was performed on the PHF13-dependent enhancers and identified motif enrichment of AP-1 members FOSL2, JUN, and FRA2, among others (Fig. 7B and Supplementary Fig. 6D). This finding was further confirmed by mapping the given regions to the publicly available transcription factor binding source [47] (Fig. 7C). Interestingly, consistent with recent findings that TGFβ broadly activates AP1-dependent enhancers via upregulating the expression of AP1 family members [5], we observed that the expression of AP1 members (*FOSL2*, *JUNB*, *ATF3*, and *ATF6*) were increased by TGFβ treatment in Panc-1 cells (Supplementary Fig. 6E). Significantly, the mRNA levels of most AP1 members (*JUNB, ATF3, FOSL1, ATF5, JUND, JDP2, ATF1, ATF6*, and *BATF3*) were reduced upon PHF13 depletion (Supplementary Fig. 6E). Together, these data indicate that PHF13 controls the AP1-dependent enhancers via facilitating AP1 family gene expression, thereby suggesting that PHF13 plays a role in regulating AP1-dependent enhancer-mediated genes in TGFβ-mediated EMT.

Given the essential role of super-enhancers (SEs) in cancer metastasis, we next sought to examine the role of PHF13 in controlling SEs during TGFβ-induced EMT. Specifically, 594 SEs were distinguished from typical enhancers via ROSE analysis of H3K27ac density [17] (Supplementary Table 3). Upon TGFβ stimulation, most SEs showed increased H3K27ac occupancy in Panc-1, whereas the occupancy of H3K27ac was decreased in most SEs in PHF13-depleted and TGFβ-treated cells compared to cells with TGFβ treatment alone (Supplementary Fig. 6F). We further found that enhancers showing differential occupancy of H3K27ac were enriched at SEs in siNC_TGFβ versus siNC and siPHF13_TGFβ versus siNC_TGFβ conditions, but not in siPHF13_TGFβ versus siNC condition (Fig. 7D). In addition, the changes in H3K27ac occupancy were much stronger in SEs than in TEs (Fig. 7E). K-means clustering and genome-wide pairwise comparison further revealed a decreased occupancy of H3K27ac at a large number of TGFβ-activated SEs in PHF13-depleted TGFβ-treated cells compared to cells with TGFβ treatment alone (Fig. 7F). Consistent with the alteration in chromatin activity, these SE-linked genes were significantly downregulated following PHF13 depletion (Supplementary Fig. 6G). Interestingly, the PHF13-dependent SEs were enriched in gene sets associated with cell metastasis, such as the extracellular matrix (Fig. 7G). Ranking the levels of the H3K27ac signal revealed that TGFβ-activated SEs were linked to the EMT-associated genes *MMP17*, *ITGA2*, *COL1A1*, and *SOX9* (Fig. 7H). This effect was confirmed at individual PHF13-dependent TGFβ-activated gene *SOX9* (Fig. 7I) and provides further support that the activation of a subset of SEs in TGFβ-induced EMT is highly dependent on PHF13.

## Discussion

Whole-genome sequencing of primary and metastatic tumors in the same patients has revealed limited heterogeneity of known driver mutations among all subclones [48]. Therefore, epigenetic alterations have been suggested as a pivotal factor in tumor metastasis [21, 49, 50]. Here, we showed that PHF13, a newly identified epigenetic reader of H3K4me2/3 [31, 32], is highly expressed in human pancreatic tumors characterized by metastasis. Utilizing a cell culture-based approach, we provide evidence that depletion of PHF13 impaired TGFβ-induced EMT in pancreas ductal adenocarcinoma cell Panc-1. Mechanistically, integrated analysis of transcriptome and epigenetic data suggest that PHF13 acts at a subset of poised promoters, broad H3K4me3 domains, and SEs and is required for the expression of a subset of TGFβ-activated genes.

EZH2, a subunit of PRC2, shows context-dependent oncogenic and tumor suppressor functions [51–55]. For example, EZH2 could promote tumor progression by interacting with SNAI1 and being driven to the promoter of E-cadherin, thus suppressing gene expression [56]. In contrast, it reduces chemotactic cell invasion via PRC2-mediated chromatin repression at the oncogene CXCR4 [57]. Interestingly, consistent with a previous study [31], we observed a strong interaction between PHF13 and EZH2. Notably, ChIP profiles of PHF13 and EZH2 revealed the co-localization of PHF13 and EZH2 at a subset of poised chromatin regions. Subsequentially, Our data show that the activation of TGFβ-dependent poised genes was impaired following PHF13 depletion, displaying increased occupancy of repressive histone modification H3K27me3 and decreased occupancy of active histone modifications H3K4me3 and H3K27ac in comparison to cells treated with TGFβ alone. These findings support the hypothesis that PHF13 antagonizes the catalytic activity of EZH2 on H3K27me3 to activate TGFβ-stimulated poised genes.

Broad H3K4me3 domains are tightly linked to genes controlling cell identity [10, 11, 44] and mark oncogenes in tumor cells [6, 7] or tumor suppressor genes [6] in normal cells [8]. Moreover, alterations in the width of H3K4me3 peaks on oncogenes in different tumor cells are associated with dysregulated transcription [6]. We further determined that TGFβ treatment in two different cell lines results in the widespread broadening of H3K4me3 peaks on metastasis-associated genes. Interestingly, the stabilization of the broad H3K4me3 domain stimulated by TGFβ was much more dependent on PHF13 than the typical H3K4me3-occupied regions. Following the depletion of PHF13, the length of TGFβ-triggered broad H3K4me3 domains was significantly narrowed. Thus, we suggest the epigenetic reader of H3K4me3 PHF13 recognizes and recruits to H3K4me3 to maintain TGFβ-induced broad H3K4me3 domains. Notably, this effect has been confirmed in the central EMT-TF SNAI1. In addition, depletion of PHF13 downregulated SNAI1-dependent genes, such as *MMP2* and *MMP9*. Thus, a novel epigenetic regulator pathway has been hypothesized to explain PHF13-mediated cancer-cell invasion, in which the H3K4me3 reader PHF13 stabilizes the TGFβ-activated broad H3K4me3 domain to maintain the highly transcribed level of *SNAI1*, thereby triggers a transcriptional program leading to EMT.

In conclusion, we have provided mechanistic insights to explain the role of PHF13 in cancer metastasis by employing genome-wide analysis to understand its effects on chromatin activity and gene expression during TGFβ-induced EMT. Furthermore, our data indicate that PHF13 is required for TGFβ to endorse EMT-related oncogene expression by stabilizing the broad H3K4me3 domains and SEs. Interestingly, PHF13 may have diverse molecular functions in gene transcription. In addition to cooperating with H3K4me2/3 to increase chromatin activity [31], PHF13 interacts with H3K9 KMTs (G9A, GLP, and SetDB1) to mediate H3K9me3-dependent chromatin condensation [29]. Notably, G9a, which shows elevated expression in multiple human cancers, plays an essential role in tumor metastasis [58]. Moreover, ChIP-seq for PHF13 in our study showed a large fragment of PHF13-bound regions not localized in chromatin assembly sites, indicating a potential role of PHF13 in the H3K9me3-marked highly compacted chromatin regions. Altogether, these findings reveal PHF13 as a novel therapeutic target for human cancers and provide a basis for further investigation of its oncogenic role in various types of human cancer.

## Materials and methods

### Cell culture, plasmids, and siRNA transfection

Panc-1 cells were kindly provided by Stem Cell Bank, Chinese Academy of Sciences. Panc-1 cells were authenticated by DNA profiling using twenty different and highly polymorphic short tandem repeat (STR) loci. Mycoplasma was also measured by PCR. Panc-1 cells were cultured in high-glucose DMEM/F12 (Thermo Fisher Scientific, New York, USA) supplemented with 10% FBS (10099, Thermo Fisher Scientific, Australia) and 1% penicillin-streptomycin (Thermo Fisher Scientific, New York, USA) at 37 °C, 5% CO_2_. For plasmids transfection, cells were transfected with pcDNA3.1(+)-PHF13 overexpressed vector using FuGENE®HD reagent (E2311, Promega, Wisconsin, USA) according to the manufacturer’s instructions. pcDNA3.1(+) empty vector was set as a negative control. siRNA transfections were performed using RNAiMAX (Thermo Fisher Scientific) according to the manufacturer’s instructions. Targeted human PHF13 siRNAs (Shanghai GenePharma Co., Ltd, China) containing the following sequences: 5′-CCCUUAUCCGAAGGAGGAATT-3′, 5′-UUCCUCCUUCGGAUAAGGGTT-3′, 5′-CCUGAUCAGGUCAAAGAAATT-3′, 5′-UUUCUUUGACCUGAUCAGGTT-3′. Non-targeting siRNA was used as a negative control (Shanghai GenePharma Co., Ltd, China). Afterward, Panc-1 cells were exposed to 10 ng/ml of TGFβ (P01137, Peprotech, USA) supplemented growth medium for 48 h to induce epithelial-mesenchymal transition.

### CRISPR/Cas9-mediated mutation of *PHF13* in Panc-1 cells

*PHF13* knockout Panc-1 cells were generated using the CRISPR/Cas9 system as described before [59]. In brief, guide RNAs targeting exon 3 of the PHF13 genes were designed using the MIT CRISPR design software and further cloned into a pSpCas9(BB)-2A-GFP (PX458) plasmids from Feng Zhang lab (Addgene plasmid #48138). According to the manufacturer’s instructions, the constructed plasmids were transfected in Panc1 cells using the X-tremeGENE HP DNA transfection reagent (XTGHP-RO, Roche, USA). GFP+ cells were obtained by sorting 48 h after plasmids transfection. Then, the single-cell clones were expanded in 96-well plates for two weeks. Finally, the positive clones were selected by genotyping and sequencing. The additional information about gRNAs and genotyping primers are listed in Supplementary Table 1.

### The pancreatic cancer xenograft mouse model

Animal experiments were performed in Zhanjun Li’s lab (Jilin University) and approved by the Institutional Animal Care and Use Committee of Jilin University. Female nude BALB/c mice (4 weeks old) were obtained from Liaoning Changsheng Biotechnology Co., Ltd. (Liaoning, China) and were randomly assigned to experimental groups, no blinding was performed. 200 µl of 2 × 10^6^ Panc1 cells were injected into the right groin of each mouse. Tumor sizes were measured and calculated as ½ (length × width^2^). After 30 days, the mice were sacrificed, and the formed tumors were weighted. Each cell line was injected into six mice.

### Western blotting, immunoprecipitation, and gene expression analysis

Protein extraction and western blot analysis were performed as previously described [60]. Cells were lysate in RIPA buffer [50 mM Tris-HCl (pH 7.4), 150 mM NaCl, 1% NP-40, 0.1% SDS] with protease inhibitors. Following sonication, protein extraction was incubated with SDS loading dye at 95 °C for 10 min. Proteins were further separated by PAGE and analyzed by western blotting using the specific antibodies that were listed in Supplementary Table 1.

Gene expression analysis was performed by quantitative real-time PCR (qRT-PCR) as described previously [60]. The primers for qRT-PCR were listed in Supplementary Table 1. In addition, the expression level of each gene was normalized to the expression of *RPLP0* and represented as fold change relative to the control cells. All experiments were performed in biological triplicate.

### Cell migration assay

Cell migratory potential was analyzed in duplicate for Panc-1 cells by transwell migration assay as described previously [61]. In addition, 20,000 cells 24 h after being treated with 10 ng/ml of TGFβ were seeded into 8.0 µm transparent PET membrane cell culture inserts (Corning, USA) and were cultured for another 48 h with TGFβ treatment. After fixation with methanol for 20 min, the migrated cells were stained with 0.1% (w/v) crystal violet diluted in PBS for 10 min. All experiments were performed in biological triplicate.

### Colony formation assay

One thousand cells were seeded in each well of a 6-well plate. The cells were cultured for 12 days and then stained with 0.1% (w/v) crystal violet diluted in PBS. The numbers of colonies in each well were counted using ImageJ software. The experiments were performed in triplicate.

### RNA sequencing (RNA-seq) and data analysis

48 h after siRNA transfection and TGFβ treatment, total RNA in Panc-1 cells was isolated using QIAzol reagent (79306, Qiagen, Germany) and quantitated using a Bioanalyzer 2100 (Agilent). The library was prepared using VAHTS mRNA-seq V3 Library Prep Kit according to the manufacturer’s protocol (NR611, Vazyme, Nanjing, China) and sequenced using the paired-end 150-bp method on the Illumina HiSeq X platform. The experiments were performed in duplicate.

The paired-end raw reads were trimmed for adaptor sequence and low-quality sequence (-q 30) using Trim Galore 0.4.1. The cleaned data were mapped on the human reference transcriptome (hg19) by STAR 2.7.0 [62]. Htseq-count 0.12.4 was used to count the reads mapped on the exon of each gene. Normalization and differential analysis of gene counts were further performed by the DESeq2 package [63]. Significant differential expressed genes were selected based on *q* value < 0.05 and the absolute value of log_2_ fold change > 0.58.

### Gene ontology (GO) and gene set enrichment (GSEA) analysis

GO enrichment analysis for selected genes was performed with the R package “clusterprofile” [64]. The significantly enriched gene terms were determined based on “p-adjust < 0.05”. Similarly, GSEA software was used to identify a set of genes in the Molecular Signatures Database (MSigDB) whose expression shows coordinately changed in response to PHF13 depletion [65].

### Chromatin immunoprecipitation (ChIP), ChIP-seqencing (ChIP-seq), and ChIP-qPCR

ChIP of histone modifications was performed as described previously [44]. 48 h after siRNA transfection and TGFβ treatment, 10^6^ Panc-1 cells in 10 cm plates were crosslinked in 1% formaldehyde for 10 min and quenched using 125 mM of Glycine for 5 min. Cells were washed using ice-cold PBS twice and lysed in nelson buffer [150 mM NaCl, 20 mM EDTA (pH 8), 50 mM Tris (pH 7.5), 0.5% NP-40, 1% Triton-X-100, 20 mM NaF]. Nuclei were pelleted and suspended in sonication buffer [150 mM NaCl, 1% NP-40, 0.5% sodium deoxycholate, 50 mM Tris-HCl, 20 mM EDTA, 20 mM NaF, 0.5% SDS]. The chromosome was fragmented in the Bioruptor® Pico via sonicating 5 cycles. The fragmented chromosome samples were diluted in two volumes of dilution buffer [150 mM NaCl, 20 mM EDTA (pH 8), 50 mM Tris (pH 8.0), 1% NP-40, 1% Triton-X-100, 20 mM NaF, and 0.50% sodium deoxycholate]. The unspecific binding sites were blocked and pulled down by incubating with 100 µl Sepharose 50% slurry for 1 h. The supernatants were incubated with each primary antibody (H3K4me3, H3K27me3, and H3K27ac) overnight at 4 °C. The DNA-protein complex was pulled down by incubating with protein A agarose beads for 1.5 h at 4 °C. The immunoprecipitated beads were washed twice with each ice-cold buffer: IP buffer [150 mM NaCl, 1% NP-40, 0.5% sodium deoxycholate, 50 mM Tris-HCl (pH 8.0), 20 mM EDTA, 20 mM NaF, 0.1% SDS], LiCl buffer [100 mM Tris-HCl (pH 8.5), 500 mM LiCl, 1% v/v NP-40, 1% w/v sodium deoxycholate, 20 mM EDTA, and 20 mM NaF], IP buffer again, TE buffer [10 mM Tris-HCl (pH 8) and 1 mM EDTA (pH 8.0)]. RNAs were degraded in 10 mM Tris-HCl (pH 8.0) buffer supplemented with 0.2 µg/µl RNase A. immunoprecipitated DNA was eluted in high SDS buffer [50 mM Tris-HCl (pH 8), 10 mM EDTA (pH 8.0), 1% SDS] supplemented with 0.2 µg/µl proteinase-K overnight at 65 °C. DNA was purified using phenol: chloroform: Isoamyl alcohol (25:24:1) and quantitated using Qubit 3.0 according to the manufacturer’s protocol (Life technologies). The experiment was performed in duplicate.

ChIP of EZH2 and PHF13 was performed as previous descriptions [66, 67]. Briefly, Panc-1 cells in one 15 cm plate were crosslinked sequentially as the followings: for 40 min in 5 mM EGS (21565, Thermo Fisher Scientific, Michigan, USA) at room temperature, for 40 min in 1% paraformaldehyde (V900894, Sigma, Missouri, USA) at 4 °C, then for 10 min in 1% formaldehyde (252549, Sigma, Missouri, USA). Then, according to the manufacturer’s protocol, ChIP was further processed using the ChIP-IT High Sensitivity Chromatin Immunoprecipitation kit (53040, Active Motif, California, USA). The rabbit polyclonal antibodies against human PHF13 for ChIP were newly designed and prepared by Wuhan ABclonal Technology company.

The sequencing library was prepared using the VAHTS Universal DNA Library Prep Kit according to the manufacturer’s protocol (ND607, Vazyme, Nanjing, China) and sequenced on the HiSeq X (Illumina) platforms.

For ChIP-qPCR, Input DNA (10% of the amount utilized for immunoprecipitation) was prepared from chromatin extracts and used for the normalization of ChIP samples. The occupancy of histone modifications was determined by RT-qPCR using the indicated primers (Supplementary Table 1), normalized to input DNA, and displayed as “% of input”.

### ChIP-seq data analysis

The quality of sequencing reads was examined with the FastQC package and ChIPQC analysis (Supplementary Table 2). Trim Galore (Version 0.4.1) was used to filter out the adaptor and low-quality reads (-q 30). The cleaned reads were aligned to the reference hg19 genome using Bowtie 2 (Version 2.4.1 [68]), with default parameters. Samtools were used to save the mapped paired reads. PCR duplicates in bam files were moved by using the Picard tool. MACS2 (Version 2.2.7.1) [69] was used to call narrow peaks for PHF13, H3K4me3, and H3K27ac with the parameters (-q 0.001) or broad peaks for EZH2 and H3K27me3 with the parameters (-q 0.1 --broad). The input sequencing data was set as a control. The height of H3K4me3 peaks was calculated using “refinepeak” in MACS2. Furthermore, the bedtool closest tool was used to calculate the distance between each peak of H3K4me3 or H3K27ac and the closest transcription start site (TSS). If the distance was less than 5000 bp, this corresponding histone modification was considered to be enriched on the genes, otherwise on a potential enhancer region.

Differential binding analysis of ChIP-seq data peaks called by MACS2 was performed using the R package “DiffBind” (Version 2.16.2 [70]). Significant differential binding peaks were determined using the DESEQ2 method with the parameter (*p* < 0.05).

The bamCoverage tool was used to generate a coverage track normalized by counts per million (CPM) (Version 3.5.0 [71]). Furthermore, the normalized score on each genomic region was called using computeMatrix. Finally, the average scores over the given genomic regions were calculated by plotProfile.

The normalized H3K27ac ChIP-seq signal was used to call super-enhancer with Rank Ordering of Super-Enhancers (ROSE) as described previously [17]. The GREAT (version 4.04) online tool was used to annotate the given enhancers on the basis of association rules: 5 kb upstream, 5 kb downstream, and 1000 kb max extension.

PHF13-bound enhancers were performed in the motif enrichment analysis. Then, the DNA sequences at the selected regions were further loaded to find motifs using HOMER (version 4.8.0) [72]. Similarly, the ReMap online tool (version 1.2) was used to determine whether a transcriptional regulator significantly co-localized with the prearranged regions [47].

## Supplementary information

Reproducibility Checklist

Supplementary Figures and Figure legends

original data of western blot

Dataset 1

Dataset 2

Dataset 3

Dataset 4

Dataset 5

## Data Availability

ATAC-seq data in Panc-1 cells were obtained from ENCODE project. H3K4me3, H3K4me1, and H3K27ac ChIP-seq data in mouse normal mammary gland NMuMG cells are available in NCBI GEO with the accession code “GSE140552”. The deep sequencing data in this study have been submitted to the NCBI GEO database with the accession numbers: GSE164824 and GSE180064.
