# Supplementary material for: PHF13 epigenetically activates TGFβ driven epithelial to mesenchymal transition
Source: Cell Death Dis. 2022 May 21;13(5):487. doi: 10.1038/s41419-022-04940-4 (PMC9124206; doi:10.1038/s41419-022-04940-4)

Supplementary Information

Figure legends

Supplementary Fig. 1 Preparation of *PHF13*-knockout Panc1 cell using CRISPR/Cas9 system. Related to Fig. 2 A western blot analysis of the protein level of PHF13 in PHF13-depleted and control Panc-1 cells. the protein ACTB was set as a loading control. **B** Crystal violet staining showing the confluency of siNT and siPHF13 Panc1 cells. Biological triplicates have been performed. **C** The model shows CRISPR/Cas9-mediated mutation of the exon 3 of the PHF13 gene. **D** PCR genotyping of the mutation on exon 3 of PHF13. **E** Sanger sequencing showing the frameshift mutation on PHF13 genes. **F** Western blot analyzed the protein level of PHF13 in wildtype and CRISPR/Cas9-mediated knockout Panc-1 cells. ACTB was set as a loading control.

Supplementary Fig. 2 Highly expressed PHF13 is related to metastasis-associated pathways in pancreatic cancers. Related to Fig. 3 A-C Heatmaps showing the differentially expressed genes based on PHF13 expression in PACA-CA (**A**), PACA-AU (**B**), and PAAD-US (**C**) projects, respectively. Those public gene expression data were available from the Canadian (PACA-CA), Australian (PACA-AU), and American (PAAD-US) groups in the International Cancer Genome Consortium (ICGC). “high” represents the top 25% of samples with the highest expression level of PHF13. “low” represents the top 25% of samples with the lowest expression level of PHF13. n, total numbers of samples. The color scale bar indicates the row-wise normalized expression signal.

D-F Dot plot showing GO results of differentially expressed genes in highly and lowly expressed PHF13 samples. The top 10 significantly enriched gene terms are shown. **G** Clustering

dendrogram of all expressed genes in the PACA-AU cohort. The dendrogram was generated based on the topological overlap. The different colors below the dendrogram indicate different gene modules identified by WGCNA. **H, I** Dot plot showing GO results of genes in green (**H**) and brown (**I**) modules. The top 15 significantly enriched gene terms are shown. **J** Morphology of Panc-1 cells following PHF13 deletion and/or treatment with TGFβ. **K and L** GSEA reveal a significant decrease in the enrichment of the TGFβ-upregulated gene set (TGFβ_Signature_UP) (**K**) and an increase in enrichment of the TGFβ-downregulated gene set (TGFβ_Signature_DOWN) (**L**) in PHF13-depleted Panc1 cells. **M** Western blot analysis of the protein level of PHF13, CDH2, and CDH1 in PHF13-KO Panc-1 cells transfected empty (negative control) and wildtype PHF13 vectors. ACTB was set as the loading control. the vectors were transfected into PHF13-KO Panc-1 cells using Lipofectamine™ 3000 Transfection Reagent (Thermo Fisher Scientific) according to the manufacturer's instructions. Afterward, cells were treated with 10 ng/ml TGFβ to induce EMT for 48 hours.

Supplementary Fig. 3 The differential occupancy of histone modifications upon TGFβ treatment or PHF13 depletion. Related to Fig. 4 **A** Venn diagram shows the overlapped number of PHF13 binding sites with accessible sites in Panc-1 cells. **B, C** Dot plot showing the differential peak calling of H3K4me3, H3K27ac, or H3K27me3 occupancy between siNC+TGFβ (NCT) and control (NC) Panc1 cells, or between siPHF13+TGFβ (PHFT) and siNC+TGFβ (NCT) Panc1 cells. The colored dots indicated significantly changed chromatin sites. A p-value less than 0.05 indicates a significant change. The occupancy of histone modifications at each peak was normalized by RPKM using the DiffBind package.

Supplementary Fig. 4 TGFβ-mediated activation of poised chromatin state is dependent on PHF13. Related to Fig. 5 A Aggregated and heatmap profiles showing the occupancy of PHF13, EZH2, H3K4me3, H3K27me3, and H3K27ac surrounding TSSs (±5kb) of TGFβ-stimulated poised genes in the control (NC), TGFβ-treated alone (NCT), and PHF13-depleted TGFβ-treated (PHFT) Panc-1 cells. All regions are sorted based on the occupancy of H3K4me3. **B** Western blots analysis showing the level of H3K27me3 in response to PHF13 depletion in HCT116 and Bel-7404 cells. TBP was set as a loading control. The down numbers indicated the normalized signal of H3K27me3. The signal of each band was measured using ImageJ. The signal of H3K27me3 was normalized to TBP and represented as fold change relative to the control cells. **C** Boxplot showing the normalized signal of H3K27me3 in control (NC), TGFβ treatment alone (NCT), and PHF13-depleted and TGFβ-treated (PHFT) cells. the H3K27me3-enriched regions were identified via MACS2 analysis with the parameters (-q 0.1 --broad). p-value was calculated using the unpaired Wilcoxon-Mann-Whitney-Test.

Supplementary Fig. 5 PHF13 is important in maintaining the TGFβ-stimulated broad H3K4me3 domain. Related to Fig. 6 A-C Distributions of significantly-regulated genes on broad, active, poised, H3K27me3-only, and neither clusters. Y-axis indicates the proportion of genes in each category. **D** Dot plots display H3K4me3 peak breadth (x-axis) versus peak height (y-axis) in siNC (NC), siNC_TGFβ (NCT), and siPHF13_TGFβ (PHFT) Panc1 cells, and identified the broad (red) and sharp (blue) H3K4me3 peaks. **E** Aggerate profiles compare the normalized occupancy of H3K4me3 and H3K27ac surrounding TSS (±5kb) of active genes in siNC, siNC_TGFβ, and siPHF13_TGFβ Panc-1 cells. **F** GSEA shows positively enriched SNAI1-suppressed and negatively enriched SNAI1-dependent gene sets upon PHF13

depletion in Panc-1 cells. The differential expressed genes upon overexpressing SNAI1 in LNCaP and LS174T cells were obtained via the online tool GEO2R. The analysis was performed between untreated and SNAI1 induced by doxycycline for 5 days in LNCaP, and between doxycycline induced for 0 hours and 48 hours in LS174T cells. **G** Genome browser showing the normalized signal of H3K27ac, H3K4me3, and mRNA-seq data at the *MMP2* locus in control, TGFβ-treated, and PHF13-depleted TGFβ-treated Panc-1. **H** Dot plots show H3K4me3 peak breadth (x-axis) versus peak height (y-axis) in control and TGFβ-treated mouse NMuMG cells. Red dots indicate the top 3% broadest H3K4me3 peaks. **I** Venn diagram shows the numbers of unique and overlapped broad H3K4me3-linked genes between control and TGFβ-treated NMuMG cells. **J** GO analysis of the 658 TGFβ-treated NMuMG-gained broad H3K4me3-linked genes. **K and L** Aggregate profiles compare the occupancy of H3K4me3, H3K4me1, and H3K27ac surrounding TSS of the 658 TGFβ-treated NMuMG gained broad H3K4me3-associated (**K**) and randomly selected (**L**) genes, in control and TGFβ-treated NMuMG cells.

Supplementary Fig. 6 Depletion of PHF13 impairs TGFβ-mediated activation at enhancers. Related to Fig. 7 **A** Dot plot showing the differential peak calling of H3K27ac occupancy on enhancer regions in siNC_TGFβ versus siNC (NCT/NC, left panel) and siPHF13_TGFβ versus siNC_TGFβ (PHFT/NCT, right panel) conditions. **B** Venn diagram showing the number of overlapped sites between PHF13-occupied regions and enhancers showing decreased occupancy of H3K27ac in response to PHF13 depletion. **C** GO analysis of PHF13-dependent enhancers linked genes. **D** The top 10 significant enrichment of known motifs on PHF13-dependent enhancers. **E** Heatmap showing the normalized mRNA counts of

AP-1 family members in control (NC), TGFβ-treated (NCT), PHF13-depleted (PHF) Panc1 cells, and TGFβ treatment in PHF13-depleted (PHFT) cells. **F** Dot plot showing the differential peak calling of H3K27ac occupancy on super-enhancers in siNC_TGFβ versus siNC (NCT/NC, left panel), siPHF13_TGFβ versus siNC_TGFβ (PHFT/NCT, middle panel), and siPHF13_TGFβ versus siNC (PHFT/NC, right panel) conditions. The red dots indicate the SE sites showing significantly changed occupancy of H3K27ac. **G** Boxplots compare SE-linked gene expression in the given conditions. p values were calculated using the paired Wilcoxon-Mann-Whitney-Test.

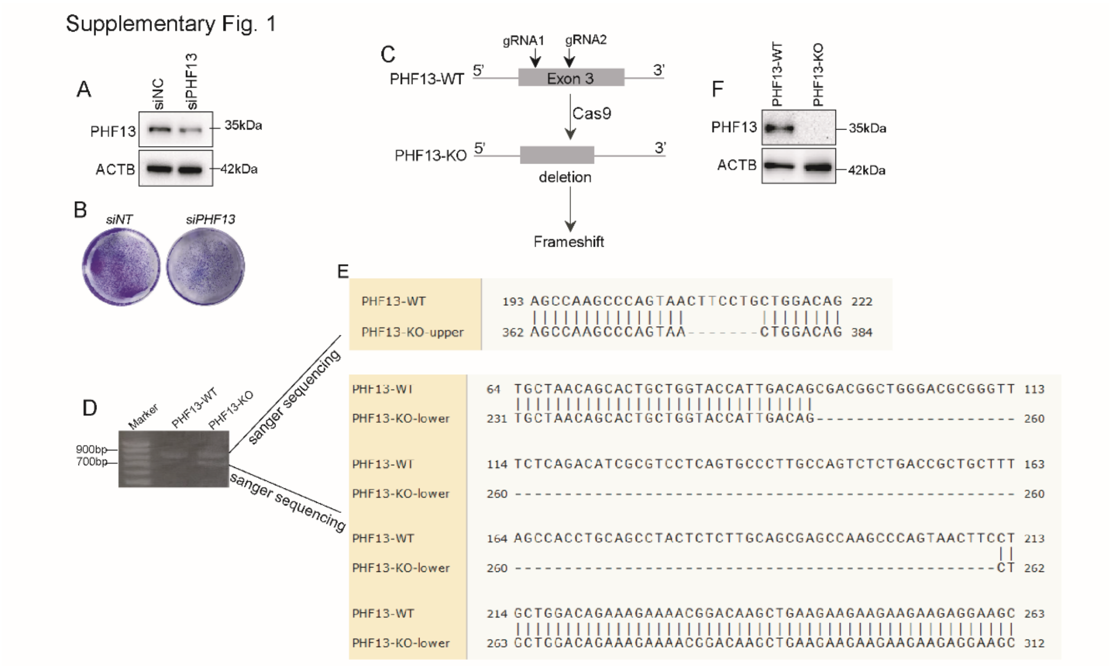

Supplementary Fig. 2

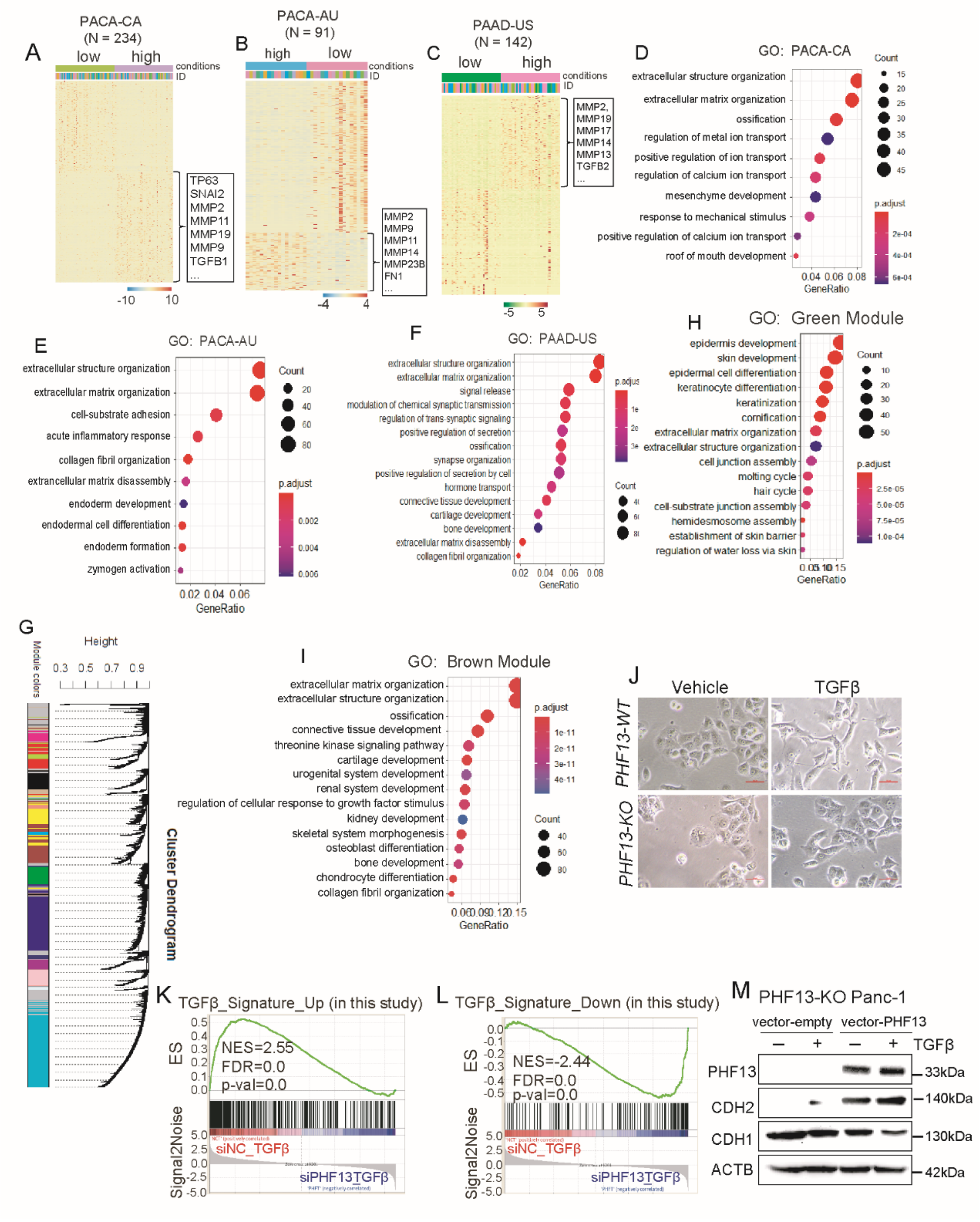

Supplementary Fig. 3

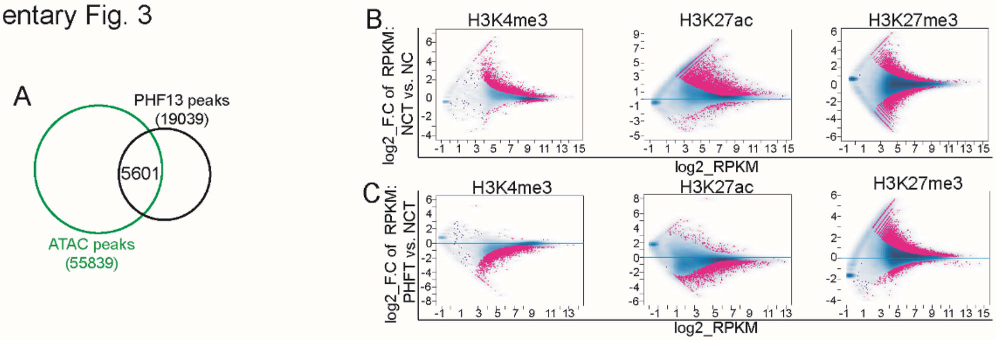

Supplementary Fig. 4

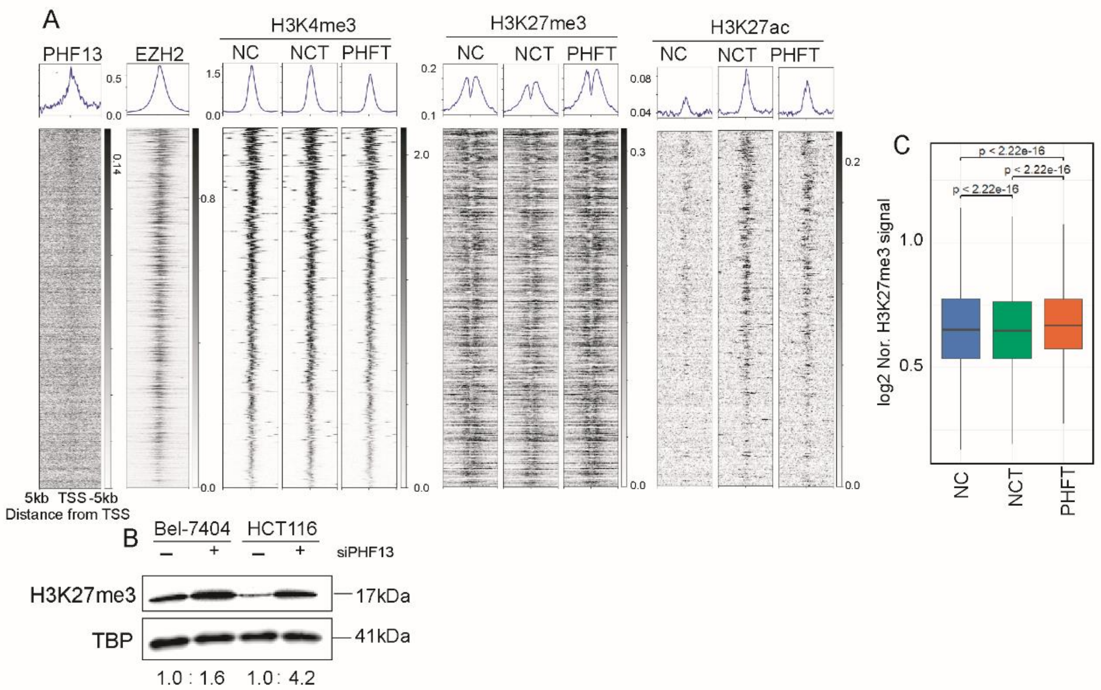

Supplementary Fig. 5

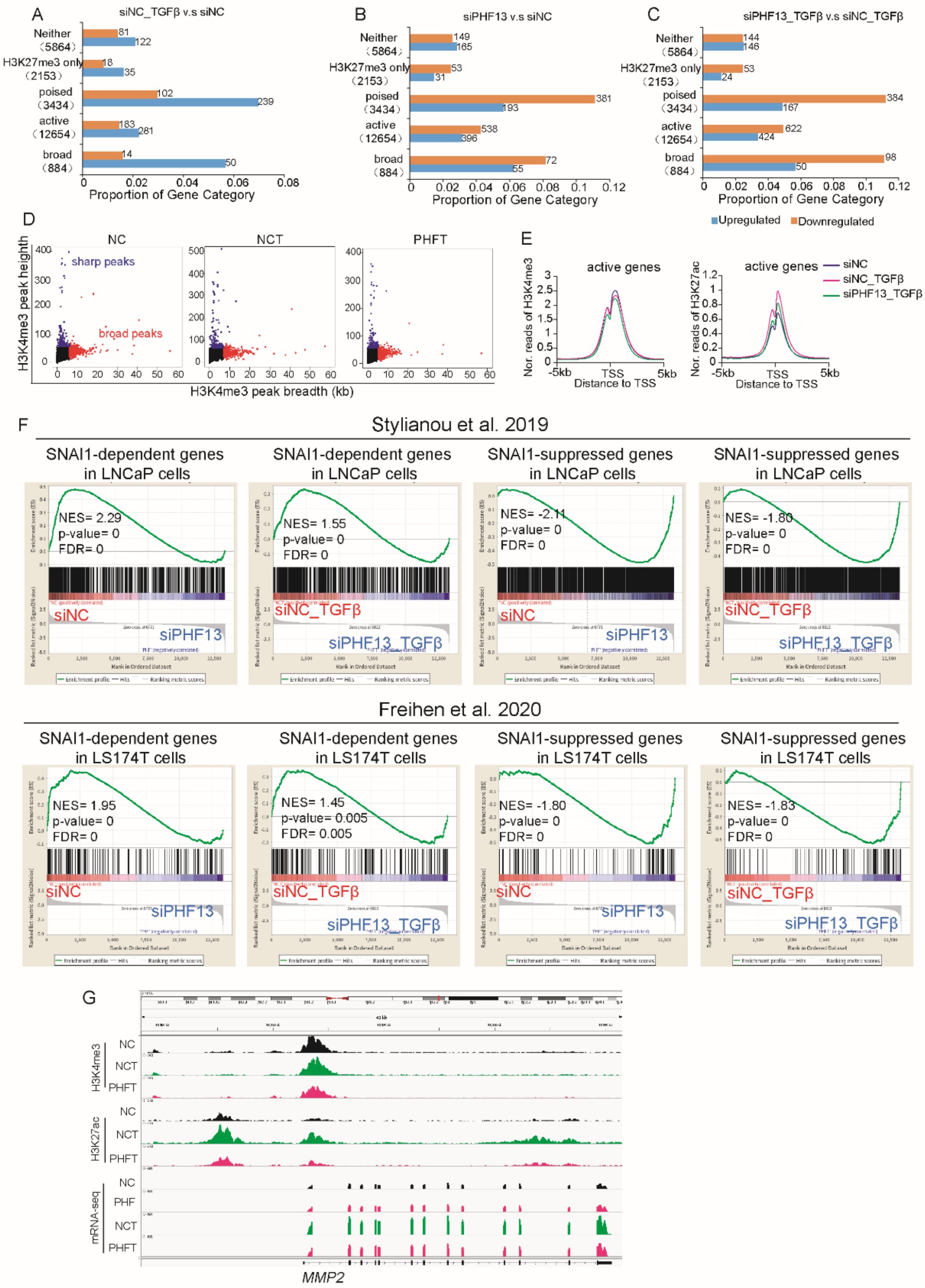

Supplementary Fig. 5

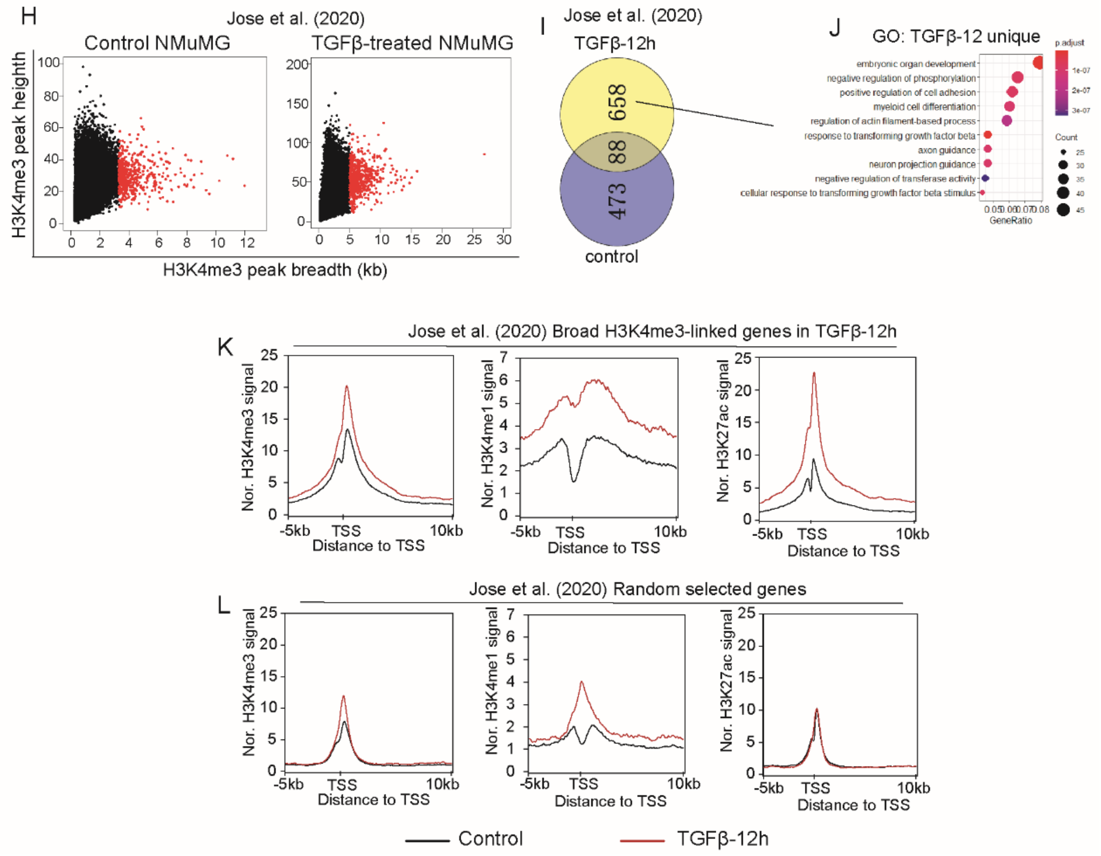

103

Supplementary Fig. 6

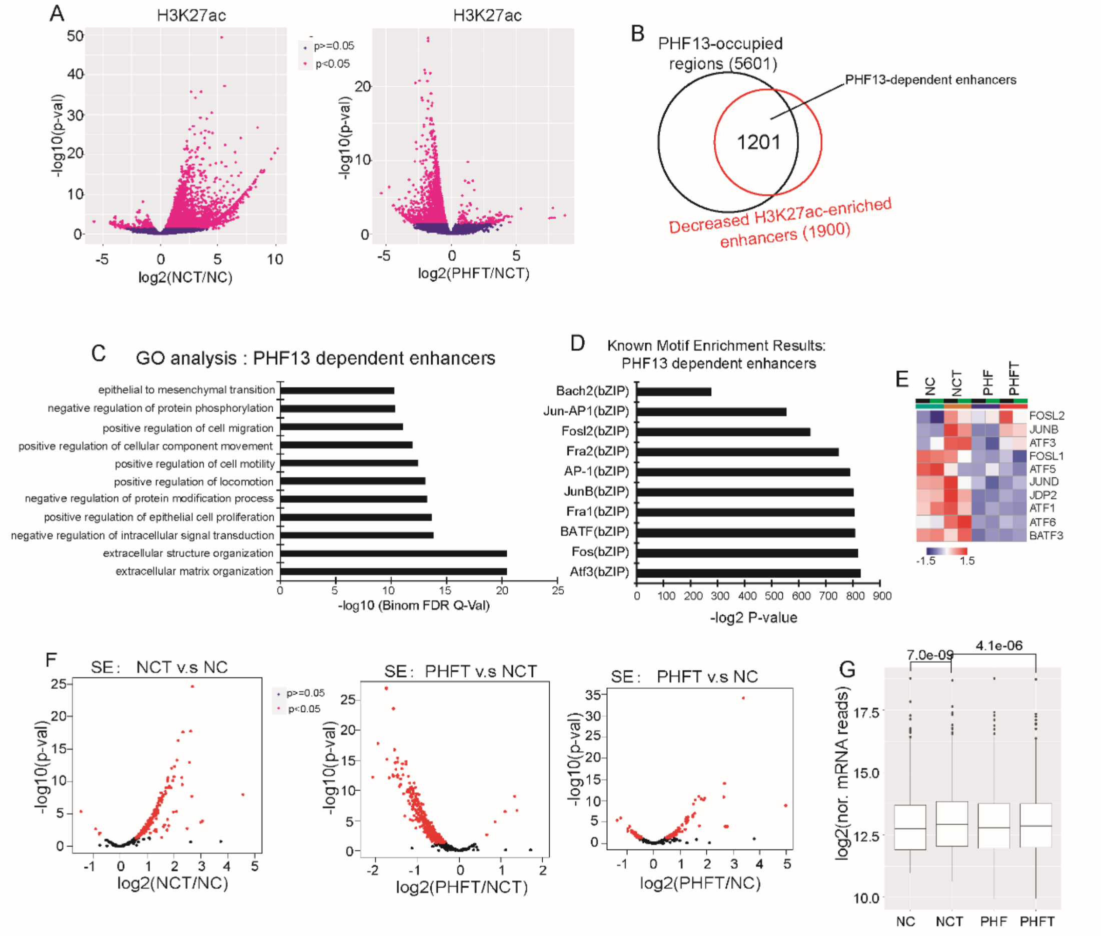

Supplement: Supplementary file 2 — Supplementary Figures and Figure legends [file 41419_2022_4940_MOESM2_ESM.pdf]
