# Supplementary material for: PHF13 epigenetically activates TGFβ driven epithelial to mesenchymal transition
Source: Cell Death Dis. 2022 May 21;13(5):487. doi: 10.1038/s41419-022-04940-4 (PMC9124206; doi:10.1038/s41419-022-04940-4)

Fig. 3K

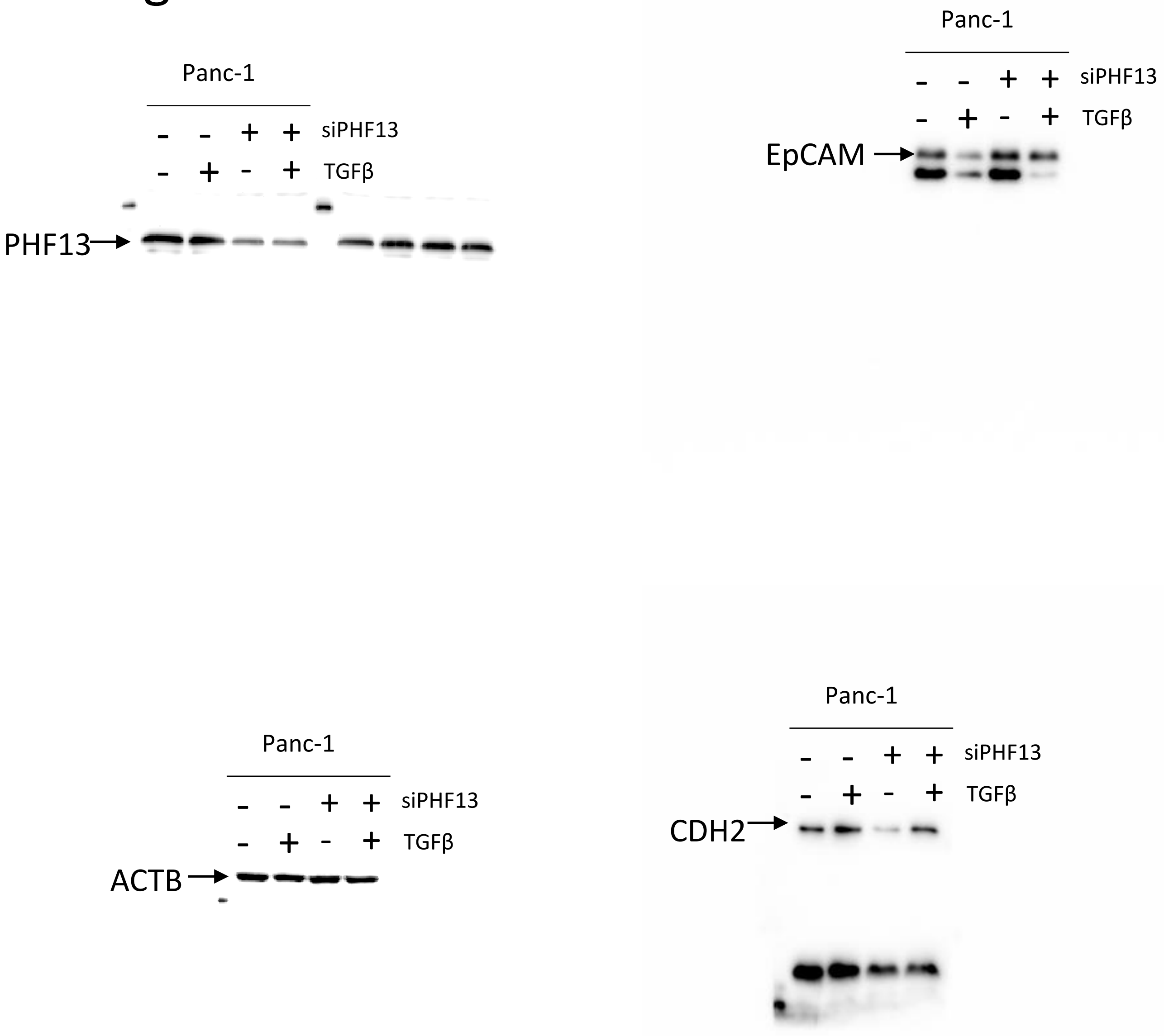
Panc-1
- - + + siPHF13
- + - + TGFβ
PHF13
Panc-1
- - + + siPHF13
- + - + TGFβ
EpCAM
Panc-1
- - + + siPHF13
- + - + TGFβ
ACTB
Panc-1
- - + + siPHF13
- + - + TGFβ
CDH2

Fig. 3M

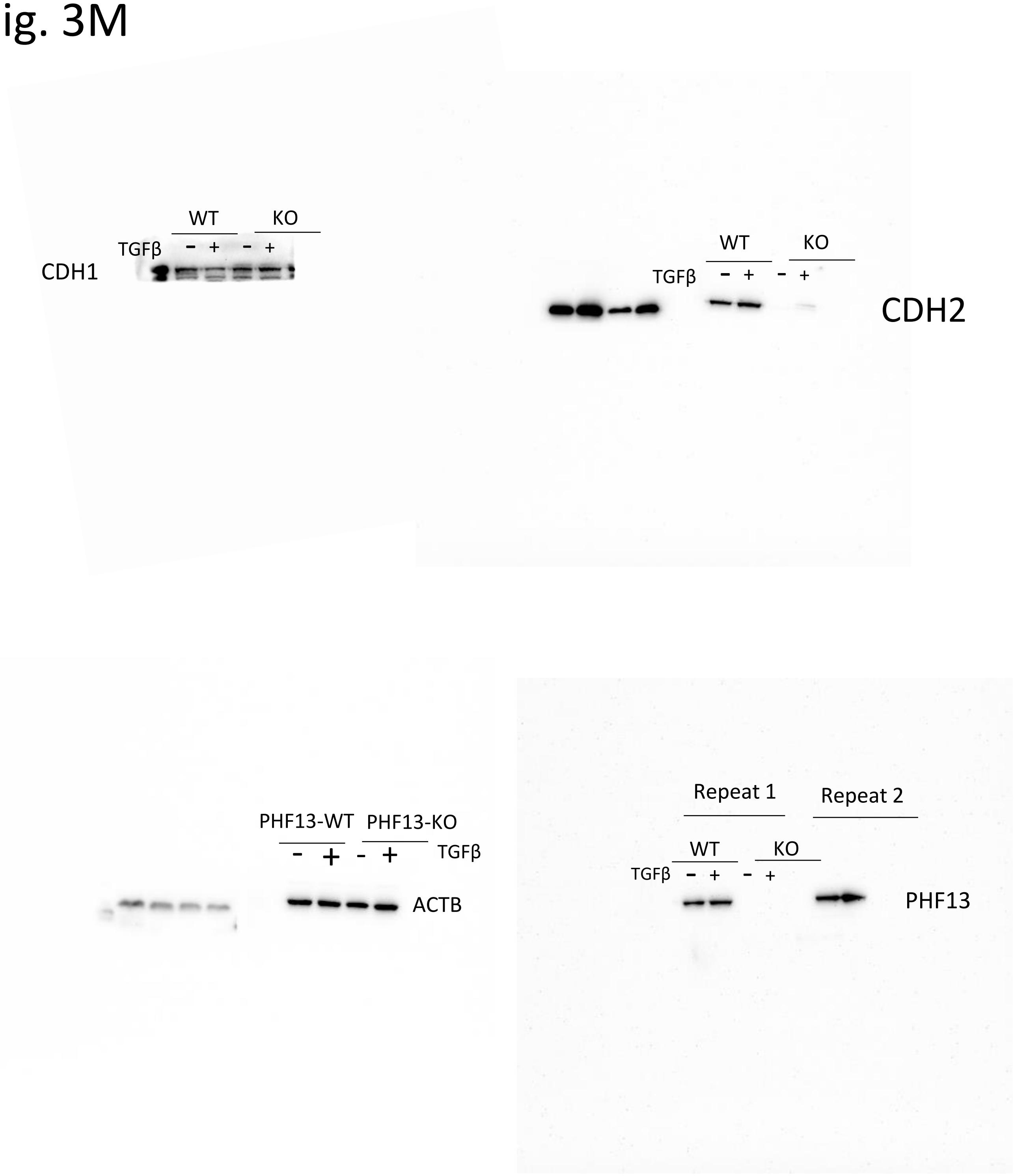

WT
KO
TGFβ
- + - +
CDH1
WT
KO
TGFβ
- + - +
CDH2
PHF13-WT
PHF13-KO
- + - +
TGFβ
ACTB
Repeat 1
Repeat 2
WT
KO
TGFβ
- + - +
PHF13

Fig. 5D

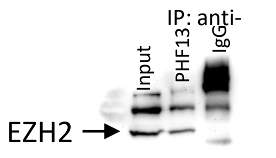

Fig. 5E

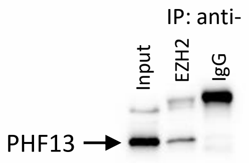

Fig. 5F

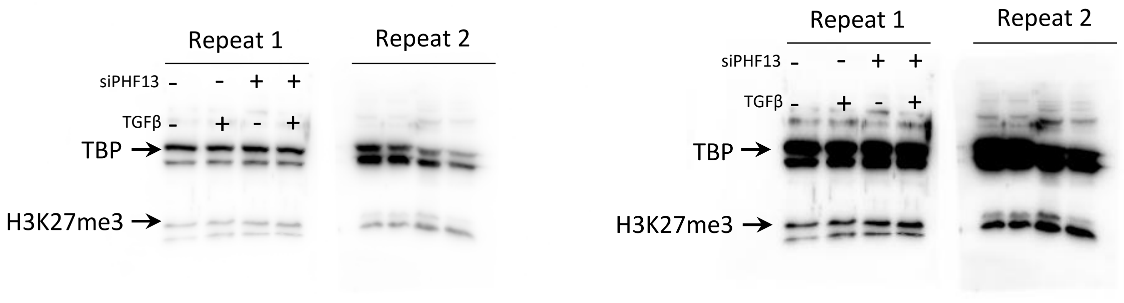

Fig. 6J

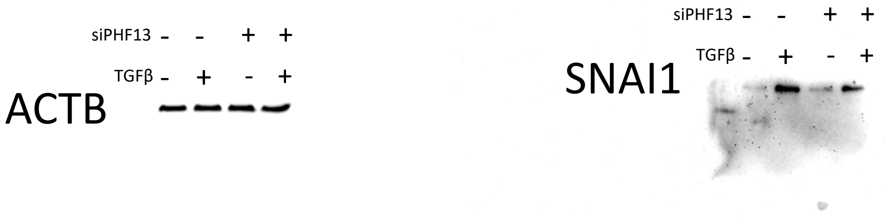

siPHF13 - - + +
TGFβ - + - +
ACTB
siPHF13 - - + +
TGFβ - + - +
SNAI1

Fig. 6K

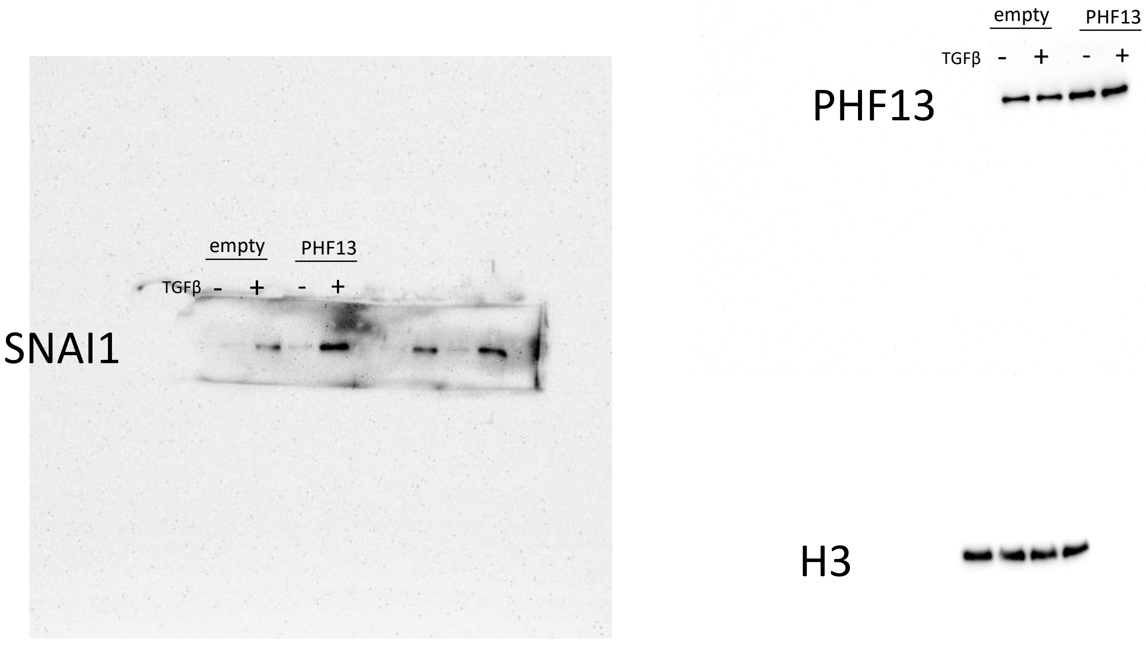

empty
PHF13
TGFβ - + - +
SNAI1
empty
PHF13
TGFβ - + - +
PHF13
H3

Supplementary Fig.2J

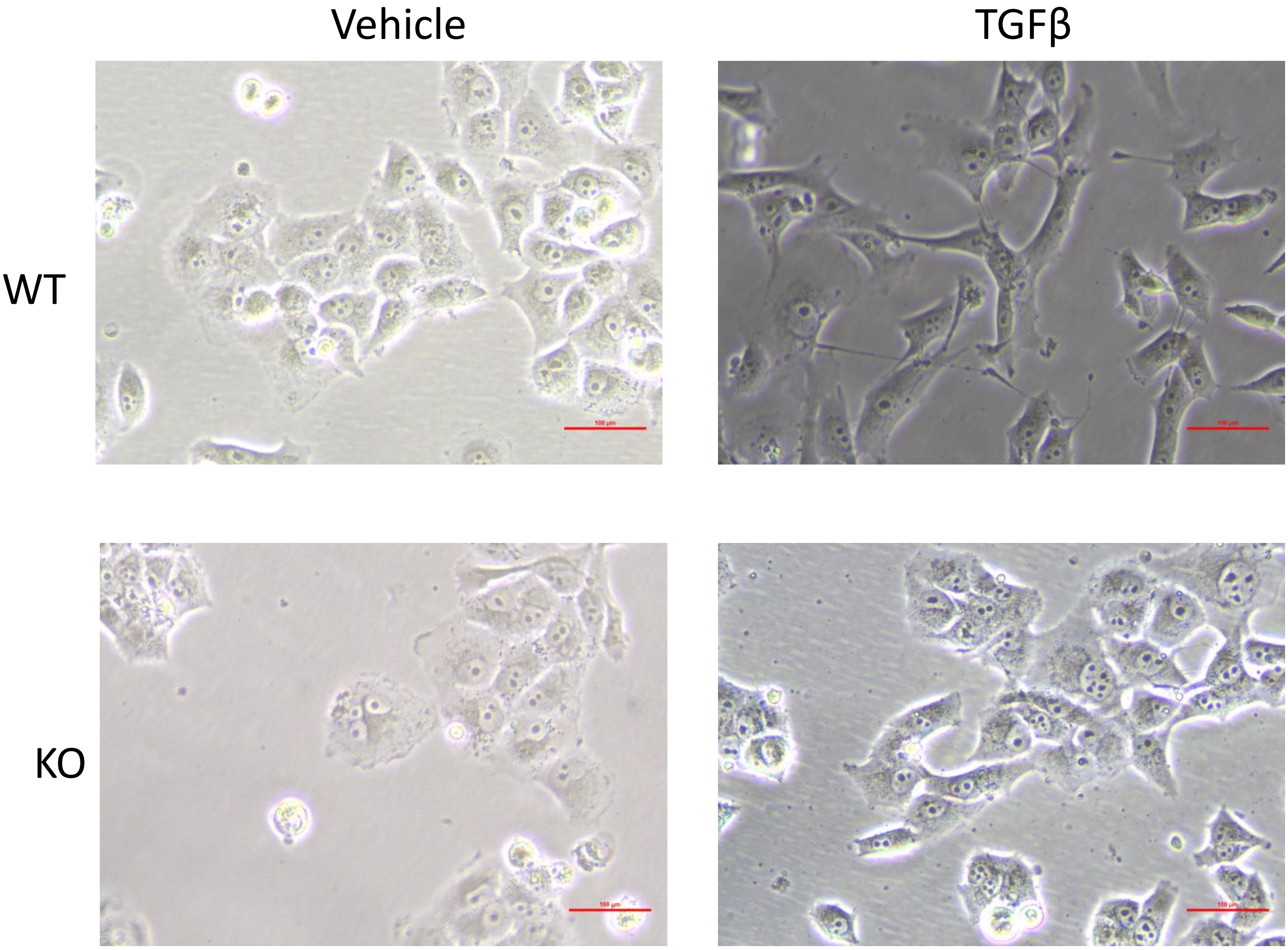

Supplementary Fig.2M

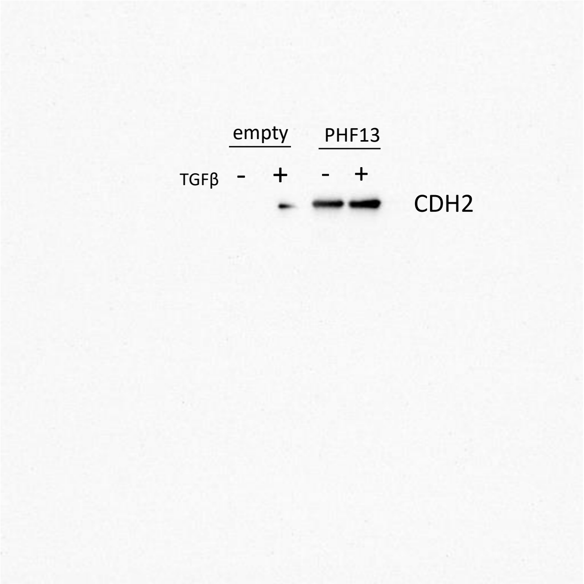

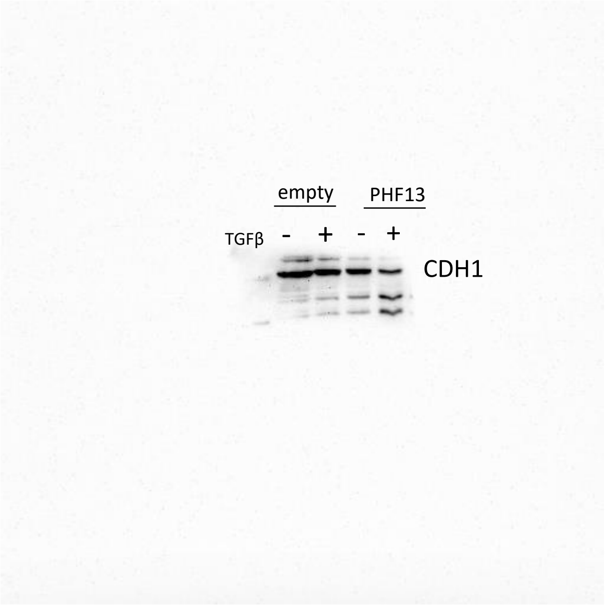

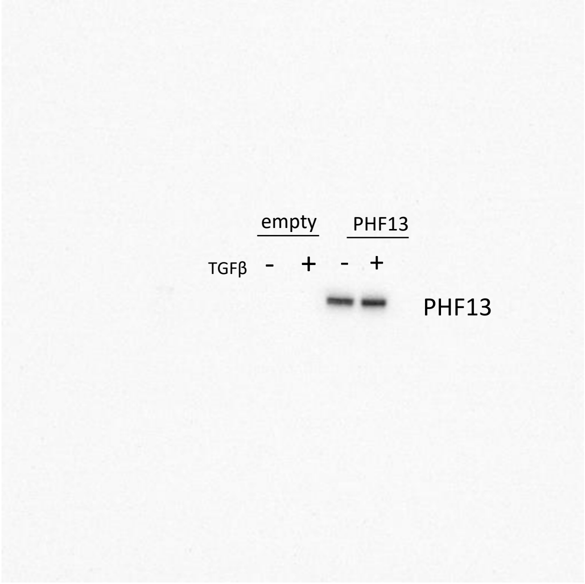

Supplementary Fig.4B

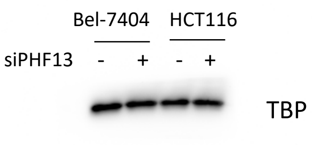

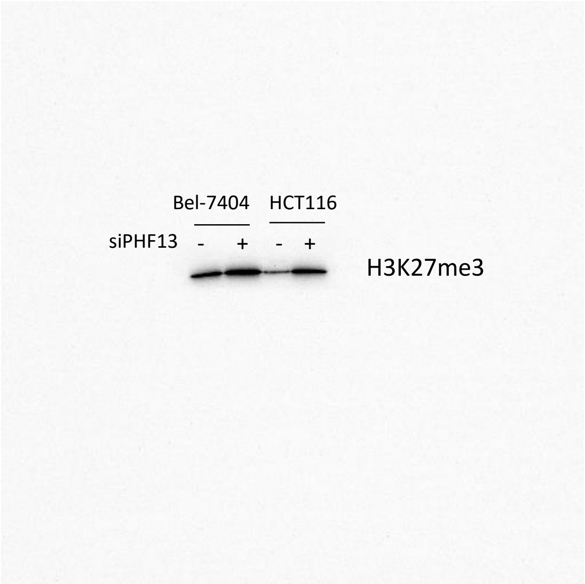

Supplement: Supplementary file 3 — original data of western blot [file 41419_2022_4940_MOESM3_ESM.pdf]
